# A chemical reporter facilitates the detection and identification of lysine HMGylation on histones†
†Electronic supplementary information (ESI) available: Experimental methods and figures. See DOI: 10.1039/c8sc02483a


**DOI:** 10.1039/c8sc02483a

**Published:** 2018-08-28

**Authors:** Xiucong Bao, Ying Xiong, Xin Li, Xiang David Li

**Affiliations:** a Department of Chemistry , The University of Hong Kong , Pokfulam Road , Hong Kong , China . Email: xiangli@hku.hk

## Abstract

Lysine 3-hydroxyl-3-methylglutarylation (HMG-K) is a newly identified PTM that can occur non-enzymatically in mitochondria. However, the substrate scope of this new PTM remains insufficiently explored, which has greatly hindered the progress in interpreting its regulatory mechanisms and cellular functions. Here, we report the development of an alkyne-functionalized chemical reporter (HMGAM-yne), for the detection and identification of cellular HMGylated proteins. HMGAM-yne is cell-permeable and metabolically incorporated into proteins in living cells. Subsequent biorthogonal conjugation enables fluorescence visualization and identification of the protein substrates of HMG-K. Using HMGAM-yne, we also identified Sirt5 as an ‘eraser’ that regulates HMGylation in cells. In addition to the known mitochondrial HMG-K proteins, HMGAM-yne facilitates the discovery of multiple nuclear proteins, including histones, as novel substrates of lysine HMGylation.

## Abstract 2

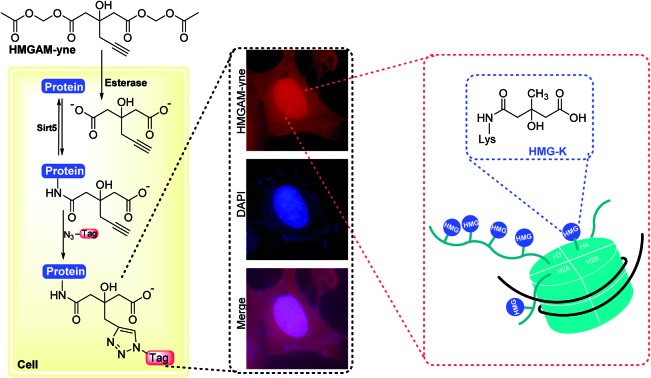
Chemical reporter, HMGAM-yne, facilitates the detection and identification of lysine HMGylation on histones.

## Introduction

Posttranslational modifications (PTMs) are involved in regulating a variety of biological processes by affecting their substrate proteins' structure, activity, cellular localization and interactions with other biomolecules. Diverse types of PTMs have been discovered, such as acetylation, methylation, phosphorylation and ubiquitinylation.^1^,^2^ To understand the cellular roles of a PTM, the essential first step is to find which proteins carry the modification. However, given the sub-stoichiometric and dynamic nature of PTMs, the detection and identification of their cellular substrates can be challenging. Commonly used methods rely on antibody-based protein enrichment coupled with mass spectrometry-based proteomics. But these methods are limited by the availability and specificity of antibodies, particularly for newly discovered PTMs. As a complementary tool, chemical reporters that are synthetic analogs of donors or donor precursors of PTMs, in conjugation with bioorthogonal chemistry, provide an alternative strategy to profile substrate proteins of PTMs. So far, chemical reporters for a wide variety of protein PTMs have been developed, including acetylation,^3^,^4^ malonylation,^5^ lipidation,^6^ methylation,^7^ glycosylation,^8^ ADP-ribosylation^9^ and AMPylation.^9^,^10^

**Fig. 1:**
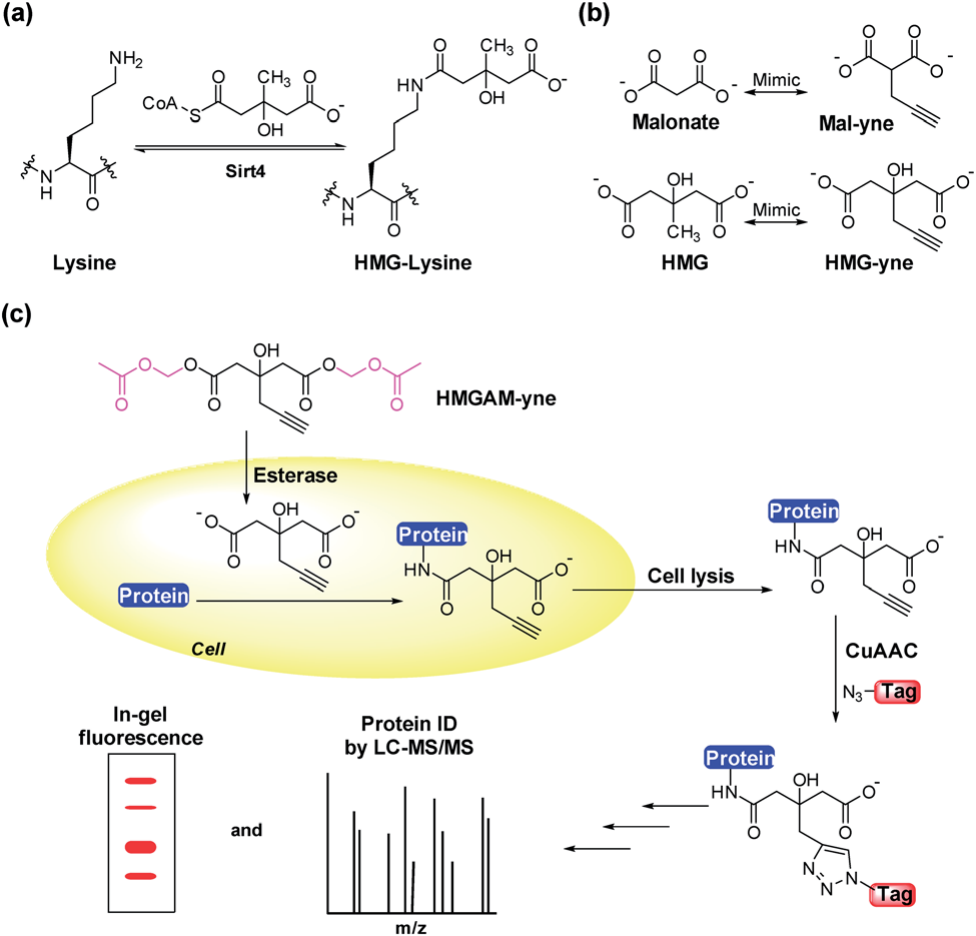
(a) The hypothesized enzymatic reactions for lysine (de)HMGylation. (b) Structures of malonate and 3-hydroxyl-3-methylglutate (HMG) and the corresponding chemical reporters, Mal-yne and HMG-yne. (c) Strategy for the visualization and identification of lysine HMGylated proteins using HMGAM-yne.

Lysine 3-hydroxyl-3-methylglutarylation (HMG-K) is a newly identified PTM that can occur non-enzymatically in mitochondria (Fig. 1a). Hirschey *et al.* discovered that negatively charged acyl-CoAs with five carbons in their acyl chains, including HMG-CoA, can effectively acylate protein lysine residues by forming highly reactive anhydride intermediates.^11^ Using a pan anti-HMG-K antibody, a number of HMGylated proteins were identified in mitochondria. The depletion of HMG-CoA lyase (HMGCL) in mouse liver, which led to the accumulation of cellular HMG-CoA, resulted in an elevated level of protein HMGylation.^11^ As the detection of HMG-K solely relied on the antibody-based approaches in this study, the substrate scope of this new PTM remains insufficiently explored, which has limited the understanding of its regulatory mechanisms and cellular functions. Here, we report the development of an alkyne-functionalized chemical reporter, HMGAM-yne, for protein HMGylation. We demonstrate that HMGAM-yne can be readily incorporated into proteins through metabolic labeling, enabling the visualization of HMGylation substrates. Pull-down experiments using this reporter not only led to the enrichment of known HMGylated proteins in mitochondria, but also the identification of multiple nuclear proteins, including histones, as the novel substrates of HMG-K.

## Results and discussion

Based on our previous work on the chemical reporter for lysine malonylation,^5^ we designed a 3-hydroxyl-3-methylglutaric acid (HMG) analogue (HMG-yne, Fig. 1b) as the reporter for lysine HMGylation. In the HMG-yne, the original methyl group was armed with a terminal alkyne, which could mediate bioorthogonal conjugation with fluorescent or affinity tags for the detection and enrichment of HMGylated proteins. At the physiological pH, the two carboxylates of the HMG are negatively charged and thus limit its permeability across the cell membrane. Therefore, we masked the two carboxylates with the acetoxymethyl (AM) group in our chemical reporter (Fig. 1c). We expected that this uncharged reporter could readily enter the cells. The cleavage of the AM esters by nonspecific cellular esterase should rapidly release the carboxylate form of the reporter inside the cells for metabolic labeling (Fig. 1c).

**Scheme 1:**
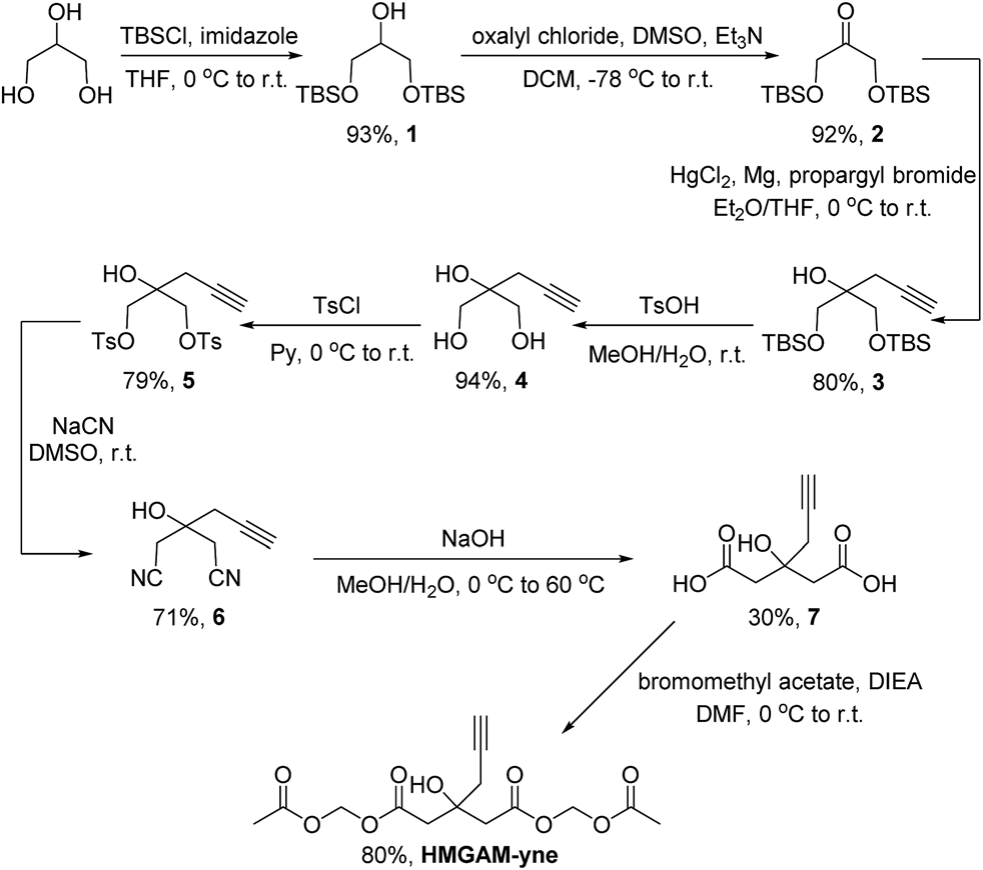
Synthetic route of HMGAM-yne. TBS = *tert*-butyldimethylsilyl, THF = tetrahydrofuran, r.t. = room temperature, DMSO = dimethyl sulfoxide, Et = ethyl, DCM = dichloromethane, Ts = tosyl, Me = methyl, Py = pyridine, DIEA = *N*,*N*-diisopropylethylamine, and DMF = *N*,*N*-dimethylformamide.

The synthesis of HMGAM-yne followed an eight-step route by using glycerol as a starting material (Scheme 1). The two primary alcohols of glycerol were first protected with *tert*-butyldimethylsilyl (TBS) ethers **1** (93% yield). The remaining secondary alcohol was then transformed into ketone **2** by mild Swern oxidation (92% yield). For the synthesis of the homopropargyl alcohol derivative **3***via* the Grignard reaction, propargylmagnesium bromide was prepared in the presence of mercury chloride at 0 °C to avoid its rearrangement into allenylmagnesium bromide. Subsequent dropwise addition of the obtained Grignard reagent to the solution of ketone **2** led to the formation of **3** (80% yield). After the deprotection of the two TBS groups, the resulting diol **4** was converted to its corresponding dinitrile **6***via* a tosylate intermediate **5** (94%, 79%, and 71% yield for each step, respectively). The following hydrolysis of the dinitrile **6** afforded the dicarboxylate **7** (30% yield), which was further masked by AM esters to give the desired HMGAM-yne (80% yield).

**Fig. 2:**
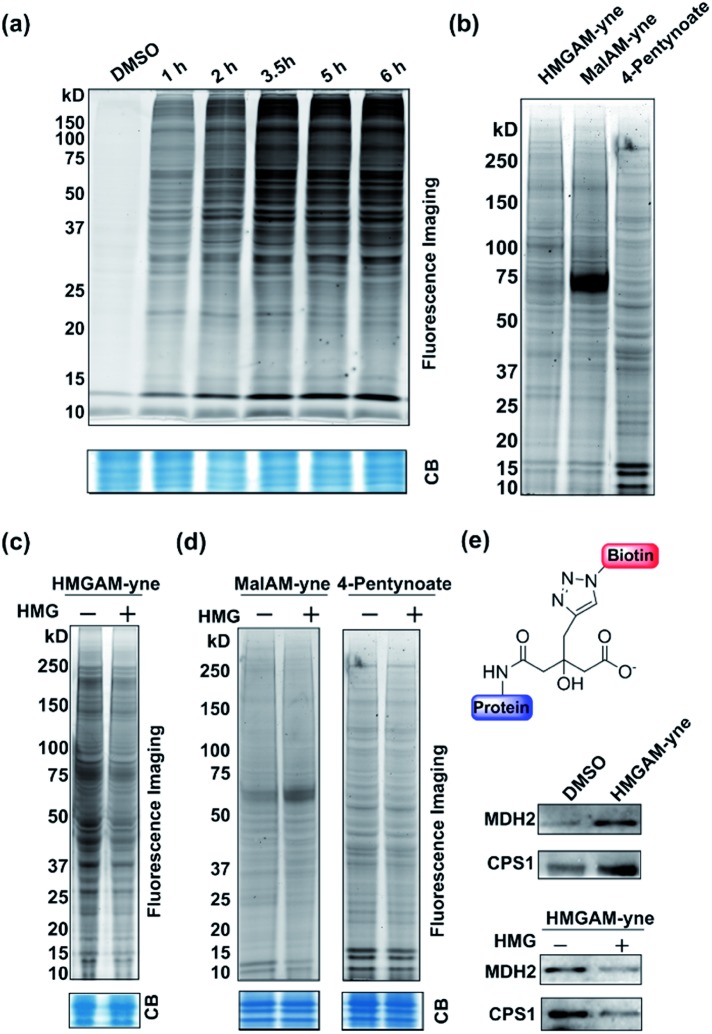
Metabolic labeling of lysine HMGylated proteins with HMGAM-yne in human cells: (a) time-dependent metabolic labeling of HeLa S3 cells with HMGAM-yne. (b) Metabolic labeling of human cells with different chemical probes, HMGAM-yne, MalAM-yne and 4-pentynoate. (c) Metabolic labeling of proteins with HMGAM-yne in the absence/presence of HMG as a competitor. (d) Metabolic labeling of proteins with chemical probes, MalAM-yne or 4-pentynoate with/without HMG as a competitor. After metabolic labeling, the labeled proteins were then conjugated to rhodamine-azide and analysed by in-gel fluorescence scanning. Coomassie-blue (CB) staining showing the equal loading. (e) Immunoblotting analyses showing the enrichment of two known protein substrates of HMGylation, CPS1 and MDH2, by HMGAM-yne.

We first examined whether HMGAM-yne could be metabolically incorporated into cellular proteins. To this end, HeLa S3 cells were treated with HMGAM-yne. After metabolic labeling, the whole-cell lysates were subjected to a Cu(i)-catalyzed azide–alkyne cycloaddition (CuAAC or ‘click’ chemistry) to conjugate the reporter-labeled proteins to a fluorescent dye (rhodamine). The labeled proteins were then resolved by SDS-PAGE and visualized by in-gel fluorescent scanning. As shown in Fig. 2a, a diverse spectrum of proteins was labeled with HMGAM-yne. The pattern of the proteins labeled with HMGAM-yne did not resemble those obtained by other tested chemical reporters for lysine acetylation (4-pentynoate^3^) and even malonylation (MalAM-yne^5^) (Fig. 2b). Importantly, the addition of HMG, a precursor of the HMG-K donor, largely impeded the labeling of proteins with HMGAM-yne (Fig. 2c), but not that with the acetylation or malonylation reporters (Fig. 2d), indicating that HMGAM-yne likely labeled HMGylation substrates. To further assess the ability of the reporter to identify HMG-K substrates, we used HMGAM-yne to enrich known HMGylated proteins. After metabolic labeling in HeLa S3 cells, the HMGAM-yne labeled proteins were conjugated to biotin and isolated using high-capacity streptavidin beads. CPS1 and MDH2, two known mitochondrial HMG-K substrates,^11^ were indeed selectively enriched by the reporter (Fig. 2e and S1†). Consistent with the fluorescent labeling experiment (Fig. 2c), the enrichment of these HMG-K substrates by HMGAM-yne was largely impaired by addition of HMG (Fig. 2e and S1†).

**Fig. 3:**
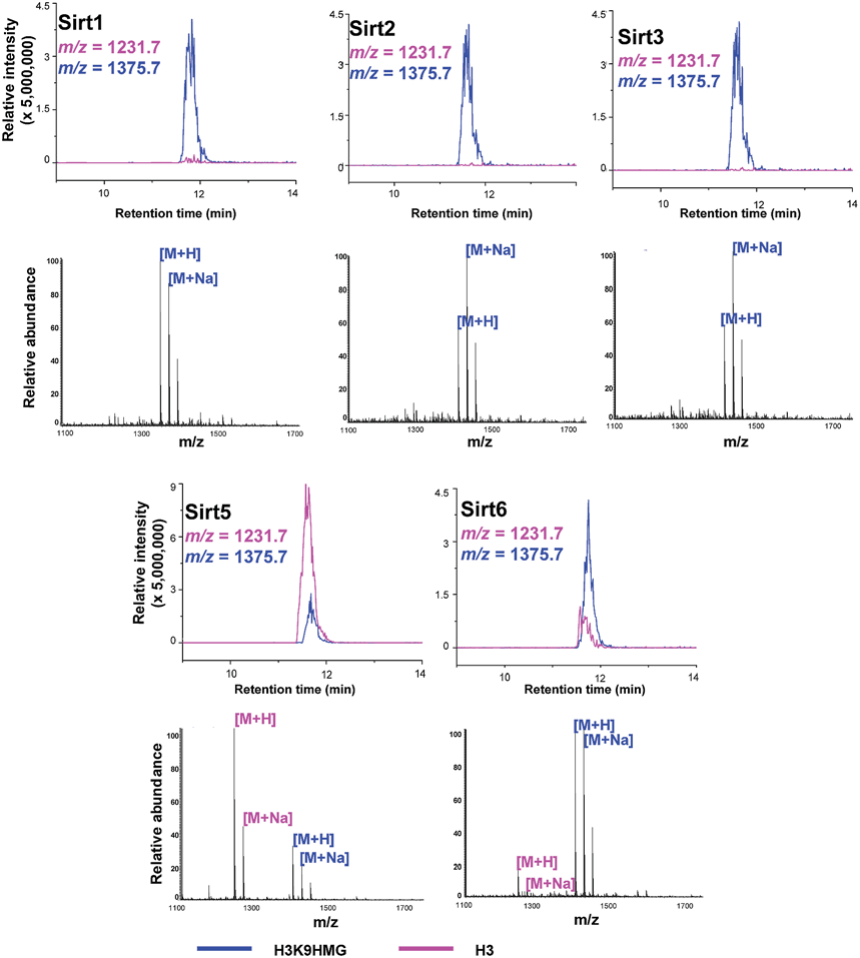
Sirtuins regulate the dynamics of lysine HMGylation. The hydrolysis of H3K9hmg by Sirts1, 2, 3, 5, and 6 was analysed by liquid chromatography-mass spectrometry (LC-MS). Pink traces show ion intensity for the masses of the de-HMGylated (unmodified) peptide, and blue traces show ion intensity for the masses of the HMGylated peptide.

**Fig. 4:**
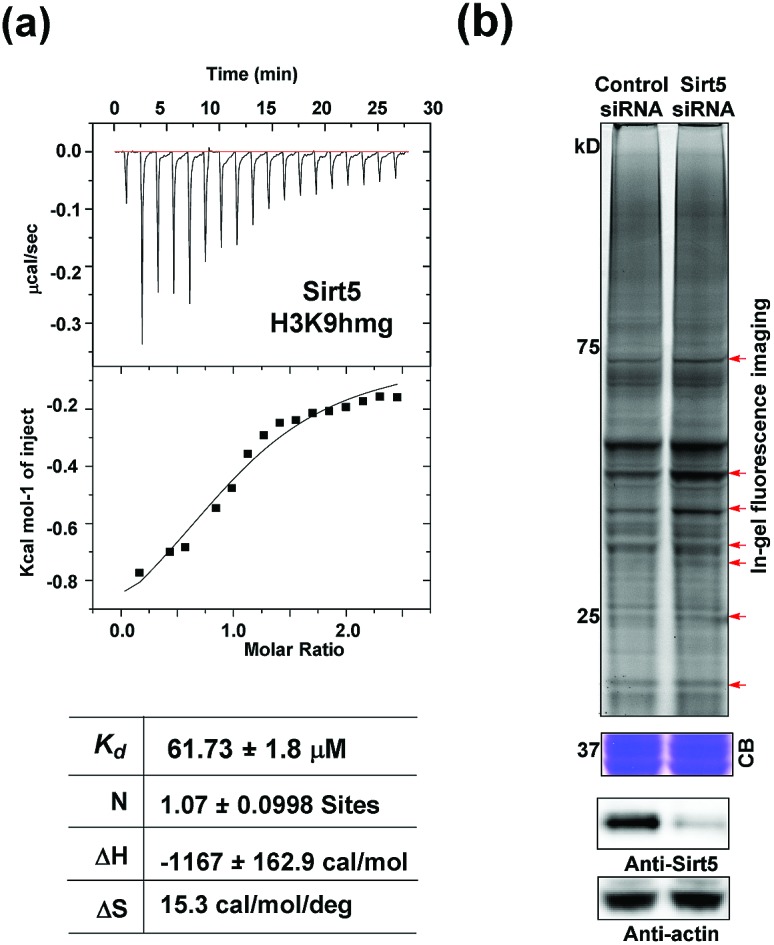
Sirt5 regulates the dynamics of lysine HMGylation: (a) isothermal titration calorimetry (ITC) measurements for the binding affinity of Sirt5 towards the H3K9hmg peptide. (b) In-gel fluorescence analyses showing that Sirt5 knockdown caused the accumulations of lysine HMGylation at some protein bands indicated with red arrows. Coomassie-blue (CB) staining showing the equal loading. Immunoblotting analysis showing the Sirt5 knockdown efficiency. γ-Actin was used as a loading control.

Like most PTMs, lysine HMGylation is a reversible process. While the addition of this modification is currently known as a non-enzymatic process, human sirtuin 4 (Sirt4) has been identified as a de-HMGylase.^12^,^13^ However, given the promiscuity of sirtuin proteins toward diverse acylations, it is possible that other members of the sirtuin family might also contribute to the regulation of cellular HMGylation. To test this hypothesis, we incubated a histone H3 peptide carrying an HMG mark at Lys9 (H3K9hmg and Note S1†), with human Sirt1, Sirt2, Sirt3, Sirt5 and Sirt6, respectively. The enzymatic reactions were then monitored by liquid chromatography-mass spectrometry (LC-MS). Among the five sirtuins tested, Sirt5 showed robust activity to catalyze the removal of HMG on H3K9 (Fig. 3). Sirt5 was recently reported to serve as the ‘erasers’ for acylations carrying a negatively charged carboxylate, including Kmal,^14^,^15^ Ksucc,^16^,^17^ and Kglu.^18^,^19^ Given the structural similarity between HMG-K and these three lysine acylations, it was not surprising that Sirt5 also has the de-HMGylase activity *in vitro*.^12^,^13^ Compared to its demalonylation and deglutarylation activities, Sirt5 exhibited a relatively low activity towards lysine HMGylation (Fig. S2a†), which may be attributed to its weak binding affinity towards the H3K9hmg peptide (*K*_d_ = 61.73 μM, Fig. 4a). For H3K9mal and H3K9glu, the *K*_d_ values were 34.97 μM and 12.55 μM, respectively (Fig. S2b and c†). We next examined whether Sirt5 can regulate protein lysine HMGylation in living cells. As shown in Fig. 4b and S3,† siRNA-induced Sirt5 knockdown resulted in the increases in the intensities of a number of protein bands labeled with HMGAM-yne, indicating that Sirt5 was involved in the regulation of the level of protein HMGylation. This is the first time that it has been shown that Sirt5 can function as an endogenous de-HMGylase in living cells.

**Fig. 5:**
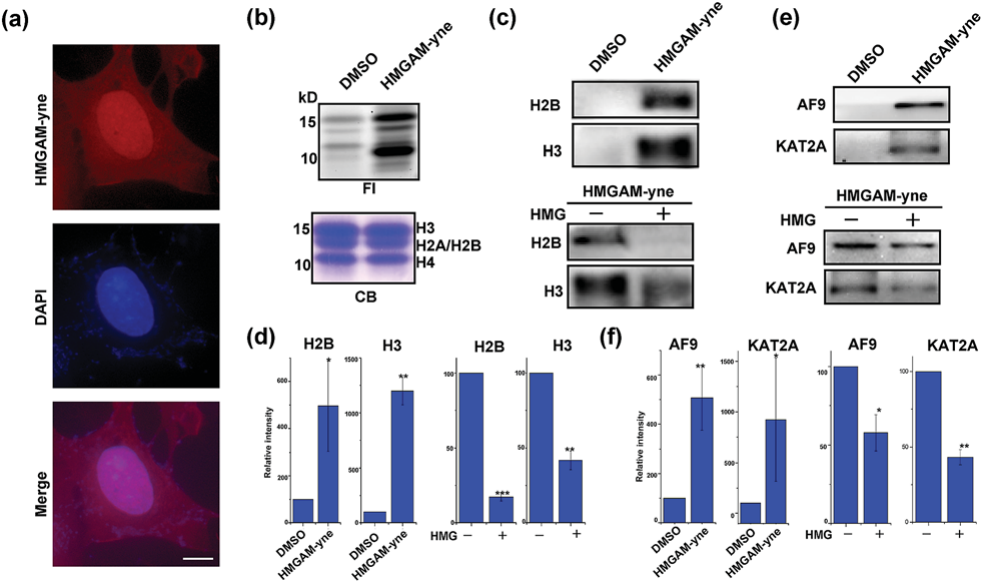
Identification of lysine HMGylation on nuclear proteins: (a) immunofluorescence analysis of cellular distribution of HMGAM-yne labeled proteins. Scale bar, 10 μm. (b) Metabolic labeling of histones using HMGAM-yne. FI: fluorescence imaging; CB: coomassie-blue staining. (c and e) Immunoblotting analysis shows the enrichment of histones ((c) H2B and H3) and other nuclear proteins ((e) AF9 and KAT2A) by HMGAM-yne. (d and f) Quantitative analysis of immunoblotting results in (c), (e), S5 and S6.† Error bars indicate ±s.e. from three independent biological replicates. The *p* values are based on Student's *t* test. *p < 0.05, **p < 0.01, ***p < 0.001.

We finally used HMGAM-yne to investigate the cellular distribution of HMGylated proteins. HeLa cells were metabolically labeled with HMGAM-yne. After fixing, the labeled proteins were conjugated with rhodamine-azide for imaging *via* fluorescence microscopy. As shown in Fig. 5a, the reporter-labeled proteins were observed in both the cytoplasm and nucleus. In line with the immunofluorescence results, in-gel fluorescence imaging of different cellular fractions also revealed that in addition to the expected fluorescence signals in mitochondria,^11^ several nuclear proteins were also labeled with HMGAM-yne (Fig. S4†). Notably, from the nuclear fraction, two protein bands labeled with HMGAM-yne had a molecular mass of approximately 15 kDa.

We speculated that the labeled proteins might be histones. By isolating histones from the cells, we indeed observed robust labeling of all the core histones with HMGAM-yne (Fig. 5b). In addition, detection against anti-H2B and anti-H3 antibodies also showed that these two histones were enriched from the pull-down sample treated with HMGAM-yne (Fig. 5c and d and S5†). Apart from the histones, we randomly selected several other nucleus-localized proteins to examine if they could also be labeled with HMGAM-yne. The results showed that, KAT2A and AF9, two epigenetic proteins associated with histone acetylation,^20^ were also likely to be the potential substrates of HMGylation as they could be specifically enriched by HMGAM-yne (Fig. 5e, f and S6†). The metabolic labeling of histones and transcriptional factors by HMGAM-yne was also significantly impeded by the addition of HMG as a competitor (Fig. 5c–f, S5 and S6†).

**Fig. 6:**
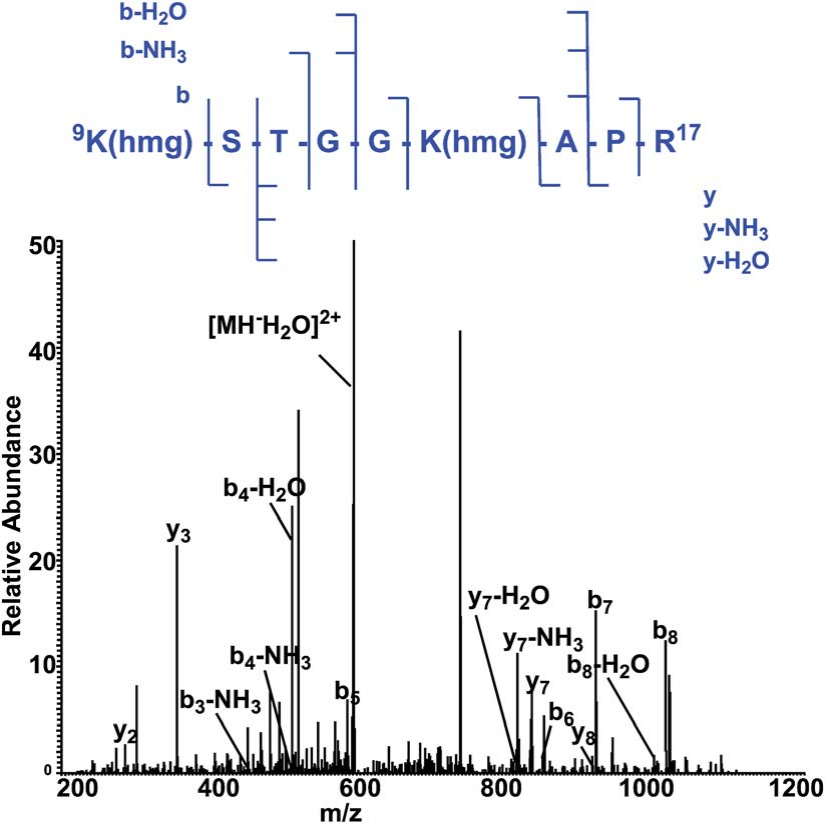
The MS/MS spectrum of a doubly charged tryptic-digest peptide, ^9^K(hmg)STGGK (hmg)-APR,^17^ from HMG-modified histone H3.

To map the modification sites of histone HMG-K, we extracted core histones from the HeLa cells for in-solution tryptic digestion and mass spectrometry (MS) analysis. The acquired MS/MS spectra were analyzed using Maxquant software to identify potential HMGylated lysine residues by setting HMG (C_6_H_8_O_4_, mass shift: +144.0423 Da) as a variable modification. We indeed identified histone peptides with a mass shift of +144.0423 Da at six lysine sites in five different histone peptides, including histone H2A Lys 119, H3 Lys9, Lys14, Lys18, Lys27, and H4 Lys59 (Fig. 6, Table S1 and Note S2†). All the MS/MS spectra of these five peptides were then manually verified to ensure the lysine residues are bona fide modification sites of HMGylation.

## Conclusions

We have developed a chemical reporter, HMGAM-yne, for the study of protein lysine HMGylation. HMGAM-yne allows for efficient metabolic labeling, robust fluorescence detection, and identification of HMGylated proteins. In addition to known HMGylated proteins in mitochondria, we also identified nuclear proteins as novel substrates, including histones. HMGAM-yne-based fluorescence detection also enabled the analysis of the dynamics of protein lysine HMGylation and identification of de-HMGylase, Sirt5. The current study will provide an important alternative method to study protein lysine HMGylation. When combined with methods to inhibit or activate the functions of HMG-regulating enzymes, our probe could be used to determine cellular substrates of de-HMGylases, such as sirtuins. Further identification of HMGylation sites and functional validation of these substrates will contribute to the comprehensive study of protein lysine HMGylation.

## Conflicts of interest

There are no conflicts to declare.

## Supplementary Material

Supplementary information
