# Supplementary material for: A chemical reporter facilitates the detection and identification of lysine HMGylation on histones
Source: Chem Sci. 2018 Aug 28;9(40):7797–801. doi: 10.1039/c8sc02483a (PMC6194501; doi:10.1039/c8sc02483a)
Supplement: Supplementary file 1 [file SC-009-C8SC02483A-s001.pdf]

Electronic Supplementary Material (ESI) for Chemical Science.
This journal is © The Royal Society of Chemistry 2018

**Supporting Information**

# Chemical Reporter Facilitates the Detection and Identification of Lysine HMGylation on Histones

Xiucong Bao, Ying Xiong, Xin Li and Xiang David Li*

Department of Chemistry, The University of Hong Kong, Pokfulam Road, Hong Kong, China.

Email: xiangli@hku.hk

## List of Contents

**General Methods and Materials**

**Scheme S1.** Chemical synthesis of HMGAM-yne.

**Scheme S2.** Chemical synthesis of Fmoc-Lys(all)-OH.

**Scheme S3**. Chemical synthesis of H3K9hmg peptide.

**Figure S1**. Metabolic labeling of MDH2 and CPS1 by HMGAM-yne.

**Figure S2**. Sirt5 regulates the dynamics of lysine malonylation, glutarylation and HMGylation.

**Figure S3**. Sirt5 regulates the dynamics of lysine HMGylation in living cell.

**Figure S4**. Metabolic labeling of mitochondrial and nuclear proteins by HMGAM-yne.

**Figure S5**. Metabolic labeling of H2B and H3 by HMGAM-yne.

**Figure S6**. Metabolic labeling of AF9 and KAT2A by HMGAM-yne.

**Table S1.** The estimated incorporation rates of HMGylated lysine in histones.

**Note S1.** Characterization of peptides**.**

**Note S2.** MS/MS spectra of lysine HMGylated peptides in histones.

**Note S3.** $^{1}$H- and $^{13}$C-NMR spectra for all the compounds synthesized in this study

**Supplementary References**

**General Methods and Materials**

Unless otherwise noted, all the chemical reagents were purchased from Sigma-Aldrich. Dulbecco's Modified Eagle Media (DMEM), pre-Stained protein, pre-case polyacrylamide gels (4-12% Bis-Tris gels) and high-capacity streptavidin beads were purchased from Thermo Scientific. EDTA-free protease inhibitor was purchased from Roche. Anti-CPS1 (sc-376190) and anti-MDH2 (sc-293474) antibodies were purchased from Santa Cruz. Anti-Sirt5 (#8782) and anti-actin (#4970S) antibodies were purchased from Cell Signaling technology. Anti-Histone H2B (ab1790), anti-Histone H3 (ab1791) and anti-KAT2A (ab18381) antibodies were purchased from Abcam. Anti-AF9 (A300-595A) was purchased from Bethyl Laboratories. Goat anti-rabbit-HRP conjugated secondary antibody and goat anti-mouse-HRP conjugated secondary antibodies were purchased from Thermo Scientific. All contrast/brightness adjustments on the images were performed in ImageJ and applied to the whole gels and blots.

All Fmoc-protected amino acids, resin for solid-phase peptide synthesis, and coupling reagents were purchased from GL Biochem. In-solution reactions were monitored by TLC silica gel 60 F254 from Merck. Flash column chromatography was performed with silica gel purchased from Grace. $^{1}H$ and $^{13}C$ NMR spectra were recorded on a Bruker UltraShield 300, 400 or 400 MHz spectrometer and were calibrated using residual undeuterated solvents as internal references. Chemical shifts were reported in values (ppm), and coupling constant J were reported in Hz. Peptides were analyzed by LC-MS with an Agilent 1260 Infinity HPLC system connected to a Thermo Finnigan LCQ DecaXP MS detector. Peptides were purified on a preparative HPLC system with Waters 2535 Quaternary Gradient Module, Waters 515 HPLC pump, Waters SFO System Fluidics Organizer, and Waters 2767 Sample Manager.

**Cell Culture**

HeLa, HeLa S3 and 293T cells were cultured in DMEM supplemented with 10% fetal bovine serum (FBS), 100 U/mL penicillin and 100 μg/mL streptomycin. Cells were maintained in a humidified 37 ℃ incubator with 5% $CO_2$.

**Metabolic Labeling and Preparation of Cell Lysates**

Cells were treated with different chemical reporters (500 μM of HMGAM-yne for 3 h, 100 μM of MalAM-yne for 1 h or 20 mM of 4-pentynoate for 6 h). DMSO (for MalAM-yne and HMGAM-yne) and PBS (for 4-pentynoate) were sued as negative controls. For competition assay, cells were preincubated with 20 mM of HMG for 6 h. Following metabolic labeling, cells were washed with ice-cold PBS trice and then lysed with ice-cold modified RIPA lysis buffer (1% NP 40, 150 mM NaCl, 50 mM HEPES, 2 mM $MgCl_2$, 10% Glycero,l pH 7.4, EDTA-free Roche protease inhibitor cocktail, 1 mM phenylmethylsulfonylfluor- ide (PMSF), 0.2U Benzonase). Cell lysates were collected after centrifuging at 16,100 *g* for 15 min at 4 ℃ to remove cell debris. Protein concentration was determined by the BCA assay (Thermo Scientific). Cell lysates were diluted with IP buffer (150 mM NaCl, 50 mM HEPES, 2 mM $MgCl_2$, 10% Glycerol, pH 7.4, EDTA-free Roche protease inhibitor cocktail) to achieve final protein concentration of 1.5 mg/mL for click chemistry reactions.

**Cu(I)-Catalyzed Cycloaddition/Click Chemistry**

Briefly, to the prepared samples, 100 μM azide-rhodamine for in-gel fluorescence scanning or cleavable azide-biotin for streptavidin enrichment was added, followed by 1 mM tris(2-carboxyethyl)phosphine (TCEP), 100 μM tris[(1-benzyl-1H-1,2,3-triazol-4-yl)methyl] amine (TBTA) and finally the reactions were initiated by the addition of 1 mM $CuSO_4$. The reactions were incubated for 1.5 h at room temperature in dark.

**In-gel Fluorescence Visualization**

The click chemistry reactions were quenched by adding 1 volume of 2× sample loading buffer. The proteins were heated at 85 ℃ for 8 min and resolved by SDS-PAGE. The labeled proteins were visualized by scanning the gel on a Typhoon 9410 variable mode imager (excitation 532 nm, emission 580 nm). Streptavidin affinity enrichment of biotinylated proteins

**Streptavidin Affinity Enrichment of Biotinylated Proteins**

HMGAM-yne-labelled cell lysate were reacted with azide-biotin (100 μM, 5 mM stock in DMSO), TCEP (1 mM, 50 mM stock in $ddH_2O$ freshly prepared), TBTA (100 μM, 10 mM stock in DMSO) and $CuSO_4 \cdot 5H_2O$ (1 mM, 50 mM stock in $ddH_2O$ freshly prepared) for 1.5 h at room temperature. Proteins were precipitated by acetone (4 volume) at -20 ℃ for overnight. Precipitated proteins were centrifuged at 3500 g for 5 min at 4 ℃ and washed with ice-cold methanol twice. To capture the biotinylated proteins by streptavidin beads, the air-dried protein pellet was dissolved in 2 mL PBS (with 20 mM EDTA, 4% SDS and 10% Glycerol) and subsequently heated at 75 ℃ for 10 min, diluted by PBS to reduce SDS concentration down to 0.5%. Pre-washed streptavidin beads were then incubated with this protein solution at room temperature for 1.5 h on end-over-end rotator. After sequentially washed six times with PBS (with 0.2% SDS), 6M Urea (in PBS with 0.1% SDS), 250 mM $NH_4HCO_3$ (with 0.05% SDS), the enriched proteins were eluted by incubating with elution buffer (25 mM $Na_2S_2O_4$, 250 mM $NH_4HCO_3$ and 0.05% SDS) for 1 h. The eluted proteins were then dried down with SpeedVac.

**Enzymatic Reactions**.

The enzymatic activities of human Sirtuins were measured by detecting the removal of HMG group from H3K9hmg peptide. 5 μM of each Sirtuin protein was incubated with 500 μM of peptide and 1 mM of nicotinamide adenine dinucleotide (NAD) in a reaction buffer containing 20 mM Tris-HCl buffer (pH 7.5) and 1 mM DTT at 37 ℃ for 2 h. The reactions were stopped by adding 1/3 reaction volume of 20% TFA and frozen in liquid $N_2$ immediately. Samples were then analyzed by LC-MS with a Vydac 218TP C18 column (4.6 mm × 250 mm, 5 μm, Grace Davison). Mobile phases used were 0.05% TFA in water (buffer A) and 0.05% TFA in ACN (buffer B). The flow rate for LC was 0.6 mL/min. The peptide mixtures were eluted by buffer A for 10 min, then 0-30% buffer B over 10 min. MS started to record at 10 min for each injection.

**Isothermal Titration Calorimetry Measurements.**

Experiments were performed at 25 ºC on a MicroCal iTC200 titration calorimeter (MicroCal). The reaction cell containing 200 μL of 150 μM proteins was titrated with 17 injections, first 0.5 μL, and all subsequent injections of 2 μL of 2.5 mM peptides. The

binding isotherm was fit with Origin 7.0 software package (OriginLab) that uses a single set of independent sites to determine the thermodynamic binding constants and stoichiometry.

**RNAi Experiments**

30 nM of Sirt5 siRNA (Thermo Scientific) was transfected into HeLa cells with Lipofectamine 2000 Transfection Reagent (Thermo Scientific), according to the manufacturer's instructions. Corresponding concentration of control siRNA was used as negative control. Following transfection, cells were then maintained in a humidified 37 ℃ incubator with 5% $CO_2$ for another 48 h.

**Immunofluorescence**

HeLa cells grown on cover slips were metabolically labeled with DMSO or HMGAM-yne, fixed with 3.7% PFA, permeabilized with 0.1% Triton X-100 and then reacted with 20 μM azide-rhodamine, 1 mM TCEP, 100μM TBTA and 1 mM $CuSO_4$ for 1 h. Cells were incubated with DAPI for 5min, washed trice with PBST (0.1% tween 20 in PBS) and then subjected to a Zeiss LSM 510 laser scanning confocal microscope.

**Cellular Fractionation**

Mitochondrial and nuclear extraction were performed as previously described [1].

**Histone Extraction**

Acid-extraction method was used to isolate histones from HeLa S3 cells [2].

**Sample Preparation for Mass Spectrometry**

In-solution tryptic digestion of histone samples was carried out based on previous described protocols [3]. Histone extracts were in-solution digested either without chemical propionylation, or chemically propionylated before or after in-solution trypsin digestion. Histone proteins or peptides were treated with propionic anhydride twice to make sure fully labeling. The final resulting peptides were enriched and desalted with the StageTips. The

eluted peptides from the StageTips were dried down by SpeedVac and then resuspended in 0.5% acetic acid for analysis by LC-MS/MS.

**Mass Spectrometry**

Mass spectrometry was performed on an LTQ-Orbitrap Velos mass spectrometer (Thermo Scientific). First, peptide samples in 0.1% formic acid were pressure loaded onto a self-packed PicoTip column (New Objective) (360-μm o.d., 75-μm i.d., 15-μm tip), packed with 7–10 cm of reverse-phase C18 material (ODS-A C18 5-μm beads from YMC), rinsed for 5 min with 0.1% formic acid and subsequently eluted with a linear gradient from 2 to 35% B in 150 min (A = 0.1% formic acid, B = 0.1% formic acid in ACN, flow rate ~ 200 nL/min) into the mass spectrometer. The instrument was operated in a data dependent mode cycling through a full scan (300–2,000 m/z, single μscan) followed by 10 CID MS/MS scans on the 10 most abundant ions from the immediate preceding full scan. The cations were isolated with a 2-Da mass window and set on a dynamic exclusion list for 60 seconds after they were first selected for MS/MS. The raw data were processed and analyzed using MaxQuant setting lysine HMGylation as a variable modification to identify H-K sites in histones. A human histone fasta file was used as protein sequence searching database. Default parameters were adapted for the protein identification and quantification.

**Synthesis of HMGAM-yne.**

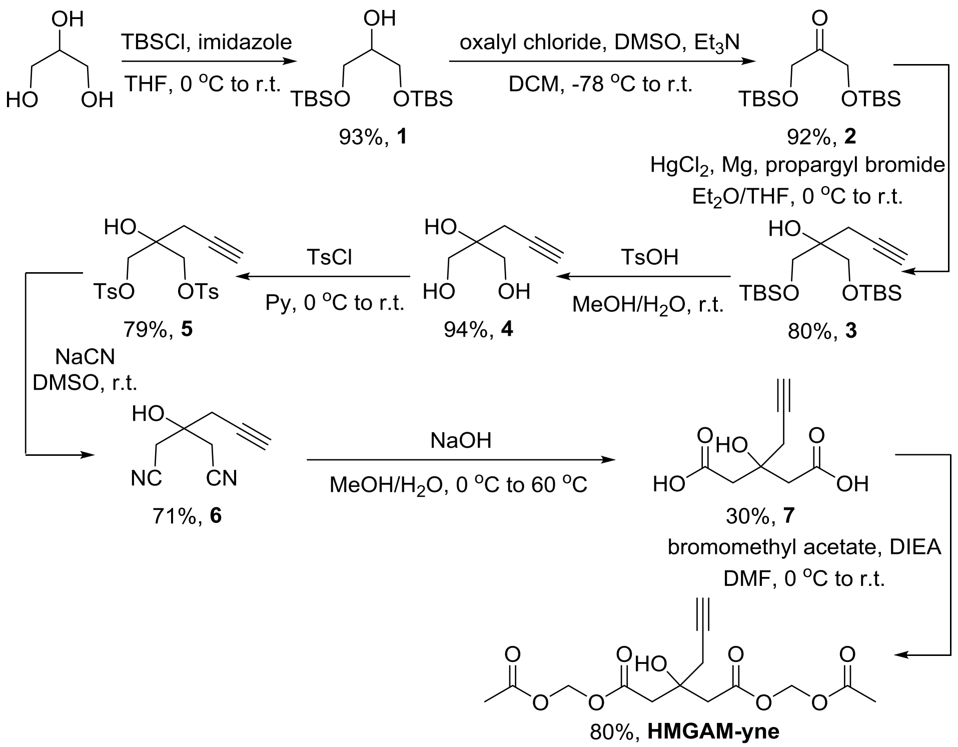


**Scheme S1.** Synthetic route of **HMGAM-yne**. TBS = *tert*-butyldimethylsilyl, THF = tetrahydrofuran, r.t. = room temperature, DMSO = dimethyl sulfoxide, Et = ethyl, DCM = dichloromethane, Ts = tosyl, Me = methyl, Py = pyridine, DIEA = *N*,*N*-diisopropylethylamine, DMF = *N*,*N*-dimethylformamide.

**Preparation of 2,2,3,3,9,9,10,10-octamethyl-4,8-dioxa-3,9-disilaundecan-6-ol (1).** To the solution of glycerol (5 g, 54 mmol) dissolved in 100 mL anhydrous tetrahydrofuran, imidazole (8.81 g, 129 mmol) and TBSCl (17.8 g, 118 mmol) were added at 0 ℃. The mixture was warmed up and stirred at room temperature for overnight. The reaction was quenched with 50 mL water and extracted with 50 mL ethyl acetate for 3 times. The organic

layers were combined and dried over anhydrous $Na_2SO_4$. After filtration and concentration, the crude product was purified by flash column chromatography (hexane:ethyl acetate = 100:1 ~ 10:1) to afford compound **1** (16.02 g 93%) as a colorless oil. $^1$H-NMR ($CDCl_3$, 400 MHz) δ 3.66–3.63 (m, 5H), 2.45 (d, *J* = 6.0 Hz, 1H), 0.89 (s, 18H), 0.07 (s, 12H). $^{13}$C-NMR ($CDCl_3$, 100 MHz) δ 71.98, 63.57, 26.00, 18.40, -5.30. The spectroscopic data match with the previous report [4].

**Preparation of 2,2,3,3,9,9,10,10-octamethyl-4,8-dioxa-3,9-disilaundecan-6-one (2).** To the solution of oxalyl chloride (4.68 g, 36.6 mmol) dissolved in 30 mL anhydrous dichloromethane at -78 ℃, dimethyl sulfoxide solution (7.3 M, 10 mL) in anhydrous dichloromethane was slowly added over 15 min. The mixture was kept stirring at -78 ℃ for 20 min and compound **1** (7.82 g, 24.4 mmol dissolved in 10 mL anhydrous dichloromethane was slowly added over 10 min. The mixture was kept stirring for another 1.5 hours at -78 ℃. Dry triethylamine (12.32 g, 122 mmol) was added and the mixture was kept stirring for 15 min at -78 ℃ before it was warmed to room temperature for another 1 hour. The reaction was quenched with 30 mL water and extracted with 30 mL ethyl acetate for 3 times. The combined organic layers were washed with 30 mL brine and dried over anhydrous $Na_2SO_4$. After filtration and concentration, the crude product was purified by flash column chromatography (hexane:ethyl acetate = 100:1 ~ 10:1) to afford compound **2** (7.62 g 98%) as colorless oil. $^1$H-NMR ($CDCl_3$, 400 MHz) δ 4.42 (s, 4H), 0.92 (s, 18H), 0.09 (s, 12H). $^{13}$C-NMR ($CDCl_3$, 100 MHz) δ 208.72, 68.00, 25.86, 18.42, -5.45. The spectroscopic data match with the previous report[5].

**Preparation of 2,2,3,3,9,9,10,10-octamethyl-6-(prop-2-yn-1-yl)-4,8-dioxa-3,9-disilaundecan-6-ol (3).** To a 50 mL flame-dried three-neck flask, mercury chloride (59 mg, 0.22 mmol), Mg (115 mg, 4.8 mmol) and one particle of iodine were added under the protection of inert gas. 10 mL dry $Et_2O$ and 10% of propargyl bromide (46.6 μL, 0.43 mmol, diluted with 1 mL dry $Et_2O$) was added at room temperature. The mixture was gently heated with hot gun till the decay of brown color in solution. The mixture was cooled to 0 ℃ with ice bath and another portion of propargyl bromide (420 μL 3.9 mmol in 3 mL dry $Et_2O$) was added over 40 minutes. Then the mixture was kept stirring at 0 ℃ for another 2 hours to afford light green supernatant with pulverized magnesium precipitates. The supernatant was used *in situ* directly.

To a solution of compound **2** (800 mg, 2.31 mmol) dissolved in 5 mL dry THF, the supernatant of the above Grignard reagent was slowly added at 0 ℃ under Ar protection. The mixture was stirred at 0 ℃ for 1 hour and gradually warmed up to room temperature for another 1 hour. The reaction was quenched with 20 mL saturated $NH_4Cl$ solution and the aqueous layer was extracted with 30 mL ethyl acetate for 3 times. The organic layers were pooled and washed with 30 mL brine, dried over anhydrous $Na_2SO_4$. After filtration and concentration, the crude product was purified by flash column chromatography to afford compound **3** (892 mg, 80%) as colorless oil. $^1$H-NMR ($CDCl_3$, 400 MHz) δ 3.61−3.52 (m, 4H), 2.69 (s, 1H), 2.41 (d, $J$ = 2.7 Hz, 2H), 2.00 (t, $J$ = 2.6 Hz, 1H), 0.89 (s, 18H), 0.06 (s, 12H). $^{13}$C-NMR ($CDCl_3$, 100 MHz) δ 80.47, 73.67, 70.55, 64.79, 25.97, 24.40, 18.35, -5.39. HRMS (EI) calculated $m/z$ for $[M]^+$: 301.1655, found: 301.1659.

**Preparation of 2-(prop-2-yn-1-yl)propane-1,2,3-triol (4).** To a solution of compound **3** (4.44 g, 12.4 mmol) in 10 mL MeOH, *p*-toluenesulfonic acid (129 mg, 0.68 mmol) was added and followed by 1 mL $H_2O$. The resulting mixture was kept stirring at room temperature overnight. The solvent was removed *in vacuo* and the resulting residue was subjected to flash column chromatography (hexane:ethyl acetate = 1:1 ~ pure ethyl acetate) to afford compound **4** (1.51 g, 94%) as sticky oil. $^1$H-NMR (MeOD, 400 MHz) δ 3.60–3.54 (m, 4H), 2.42 (d, *J* = 2.7 Hz, 2H), 2.29–2.27 (t, *J* = 2.7 Hz, 1H). $^{13}$C-NMR (MeOD, 100 MHz) δ 81.09, 75.02, 71.68, 65.49, 24.99. HRMS (ESI) calculated *m*/*z* for $[M+Na]^+$: 153.0528 found: 153.0525.

**Preparation of 2-hydroxy-2-(prop-2-yn-1-yl)propane-1,3-diyl bis(4-methylbenzenesulfonate) (5).** To the solution of compound **4** (1.5 g, 11.6 mmol) in 50 mL anhydrous pyridine, TsCl (6.62 g, 34.89 mmol) was added slowly at 0 ℃. The resulting mixture was kept stirring at room temperature overnight and quenched with 30 mL water. The resulting aqueous mixture was extracted with 30 mL ethyl acetate for 3 times. The organic layers were combined and washed with 1 M HCl for 3 times and dried over anhydrous $Na_2SO_4$. After filtration and concentration, the resulting residue was subjected to flash chromatography (hexane:EA = 6:1) to afford compound **5** (4 g, 79%) as white solid. $^1$H-NMR ($CDCl_3$, 400 MHz) δ 7.77 (d, *J* = 8.3 Hz, 4H), 7.36 (d, *J* = 8.0 Hz, 4H), 4.01 (m, 4H), 2.61 (s, 1H), 2.46–2.44 (m, 8H), 1.97 (t, *J* = 2.7 Hz, 1H). $^{13}$C-NMR ($CDCl_3$, 100 MHz) δ 145.58, 132.11, 130.19, 128.21, 72.90, 71.44, 70.38, 25.09, 21.84. HRMS (ESI) calculated *m*/*z* for $[M+Na]^+$: 461.0705 found: 461.0701.

**Preparation of 3-hydroxy-3-(prop-2-yn-1-yl)pentanedinitrile (6).** To the solution of compound **5** (2 g, 4.57 mmol) dissolved in 5 mL anhydrous DMSO, NaCN (671 mg, 13.7 mmol) was added. The mixture was kept stirring at room temperature overnight. The reaction was quenched by 20 mL water and the aqueous layer was extracted with 20 mL ethyl acetate for 3 times. The combined organic layers were washed with 20 mL water for twice and dried over anhydrous $Na_2SO_4$. After filtration and concentration, the resulting residue was subjected to flash column (hexane:EA = 6:1) to afford compound **6** (480 mg, 71%) as yellow liquid. $^1$H-NMR ($CDCl_3$, 300 MHz) δ 2.99 (s, 1H), 2.87 (s, 4H), 2.75 (d, *J* = 2.6 Hz, 2H), 2.29 (t, *J* = 2.6 Hz, 1H). $^{13}$C-NMR ($CDCl_3$, 100 MHz) δ 116.05, 77.47, 74.23, 70.73, 29.90, 28.53. HRMS (EI) calculated *m*/*z* for $[M]^+$: 130.0531 found: 130.0522.

**Preparation of 3-hydroxy-3-(prop-2-yn-1-yl)pentanedioic acid (7).** To the solution of compound **6** (271 mg, 1.83 mmol) dissolved in 5 mL MeOH, 5 mL 35% (v/v) NaOH aqueous solution was added slowly at 0 ℃. The resulting solution was kept stirring at 60 ℃ overnight. The mixture was diluted with 20 mL ethyl acetate and the organic layer was discarded after partition. The aqueous layer was carefully acidified to pH = 1 at 0 ℃ with concentrated HCl. The aqueous solution was extracted with 30 mL ethyl acetate for 3 times and organic layers were pooled and dried over anhydrous $Na_2SO_4$. After filtration and concentration, the resulting residue was subjected to flash column chromatography (MeOH: dichloromethane = 10:1 + 1% acetic acid) to afford compound **7** (100 mg 30%) as yellow oil. $^1$H-NMR (MeOH, 300 MHz) δ 2.84–2.73 (m, 4H), 2.69 (d, *J* = 2.7 Hz, 2H), 2.37 (t, *J* = 2.7 Hz, 1H). $^{13}$C-NMR (MeOH, 100 MHz) δ 174.53, 80.93, 72.55, 71.79, 43.04, 30.91. HRMS (EI) calculated *m*/*z* for $[M]^+$: 209.0426 found: 209.0418.

**Preparation of HMGAM-yne.** To the solution of compound **7** (110 mg, 0.59 mmol) dissolved in 1 mL anhydrous DMF, bromomethyl acetate (127 μL, 1.3 mmol) was added slowly at 0 ℃ and followed by DIEA (260 μL, 1.5 mmol), the resulting mixture was kept stirring at room temperature overnight. The reaction was diluted with 20 mL ethyl acetate and washed with 10 mL water for 3 times. The organic layers were pooled and washed with 10 mL brine and dried over anhydrous $Na_2SO_4$. After filtration and concentration, the resulting residue was subjected to flash column (hexane:EA = 3:1~1:1) to afford **HMGAM-yne** (155 mg 80%) as colorless oil. $^1$H-NMR ($CDCl_3$, 400 MHz) δ 5.77 (s, 4H), 3.78 (s, 1H), 2.96–2.86 (m, 4H), 2.67(d, *J*= 2.64, 2H), 2.13–2.11 (m, 7H). $^{13}$C-NMR ($CDCl_3$, 100 MHz) δ 170.02, 169.69, 79.31, 72.36, 71.13, 41.96, 30.56, 20.80. HRMS (ESI) calculated *m*/*z* for $[M+Na]^+$: 353.0849 found: 353.0844.

**Synthesis of Fmoc-Lys(all)-OH.**

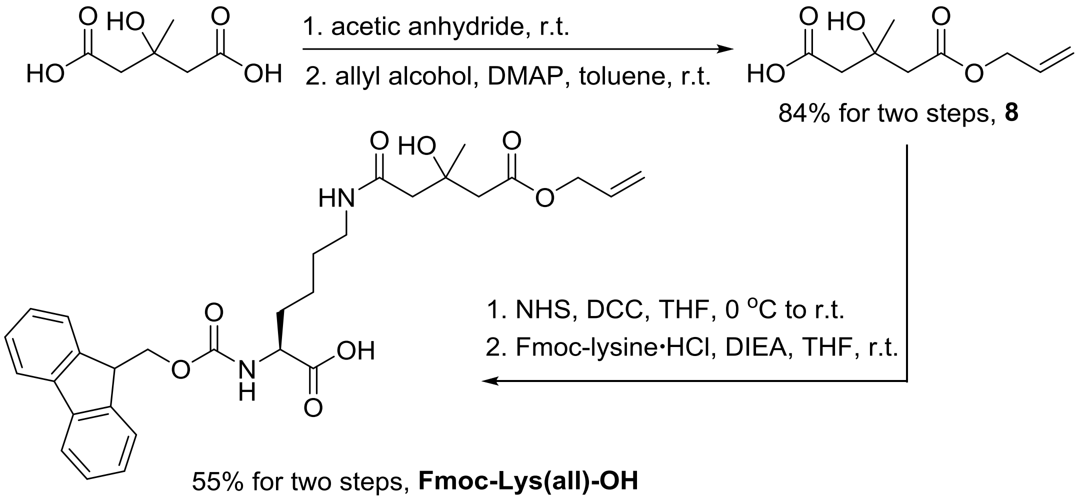


**Scheme S2.** Synthetic route of **Fmoc-Lys(all)-OH**. DMAP = 4-dimethylaminopyridine, r.t. = room temperature, NHS = *N*-hydroxysuccinimide, DCC = *N*,*N'*-

dicyclohexylcarbodiimide, THF = tetrahydrofuran, Fmoc = 9-fluorenylmethyloxycarbonyl, DIEA = *N*,*N*-diisopropylethylamine.

**Preparation of 5-(allyloxy)-3-hydroxy-3-methyl-5-oxopentanoic acid (8).** To a 25 mL round bottom flask, 3-hydroxy-3-methylglutaric acid (230 mg, 1.42 mmol) was dissolved in 6 mL acetic anhydride. The resulting solution was kept stirring at room temperature for 5 hours. The solvent was removed *in vacuo* to afford the corresponding anhydride as white solid, which was directly subjected to the following step without further purification.

To the solution of the above-obtained solid in 20 mL anhydrous toluene, allyl alcohol (482 μL, 7.1 mmol) and DMAP (17.3 mg, 0.14 mmol) were added at room temperature. The resulting mixture was kept stirring at room temperature overnight. The mixture was diluted with 20 mL ethyl acetate and washed with 10 mL water for 3 times. The organic layer was pooled and dried over anhydrous $Na_2SO_4$. After filtration and concentration, the resulting residue was subjected to flash column chromatography (EA:hexane=1:1) to afford compound **8** (242 mg, 84%) as colorless liquid. $^1$H-NMR ($CDCl_3$, 400 MHz) δ 5.93–5.88 (m, 1H), 5.36–5.26 (m, 2H), 4.64–4.63(d, *J* = 5.8 Hz) 2.76–2.64 (m, 4H), 1.41 (s, 3H). $^{13}$C-NMR (DMSO-$d_6$, 100 MHz) δ 174.85, 172.29, 133.65, 118.36, 70.68, 66.08, 46.14, 45.84, 27.72. HRMS (ESI) calculated *m/z* for $[M+Na]^+$: 225.0739 found: 225.0733.

**Preparation of Fmoc-Lys(all)-OH.** To the solution of compound **8** (448 mg, 1.2 mmol) dissolved in 10 mL anhydrous THF, NHS (157 mg, 1.37 mmol) and DCC (282 mg, 1.37 mmol ) were slowly added at 0 °C. The resulting mixture was gradually warmed to room temperature for overnight.

The resulting white suspension was filtrated through celite into a 25 mL round bottom flask. Fmoc-lysine·HCl (383 mg, 0.95 mmol) and DIEA (480 μL, 2.74 mmol) were added. The resulting mixture was stirred at room temperature for overnight. The reaction was quenched by adjusting pH to 7 with 1 M HCl at 0 ℃. Solvent was removed *in vacuo* and 5 mL $H_2O$ was added to the mixture and pH was adjusted to 2 with 1 M HCl at 0 ℃. The mixture was extracted with 30 mL dichloromethane for 3 times and the organic layer was pooled and dried over anhydrous $Na_2SO_4$. After filtration and concentration, the crude product was subjected to column chromatography (EA: hexane = 1:3 + 1% acetic acid) to afford **Fmoc-Lys(all)-OH** (287 mg, 55%) as colorless solid. $^1$H-NMR (MeOD, 500 MHz) δ 7.79 (d, *J* = 7.5 Hz, 2H), 7.67 (t, *J* = 7.9 Hz, 2H), 7.38 (t, *J* = 7.4 Hz, 2H), 7.30 (t, *J* = 7.5, 2H), 5.92 (m, 1H), 5.30 (dd, *J* = 17.2, 1.5 Hz, 1H), 5.19 (dd, *J* = 10.5, 1.3 Hz, 1H), 4.56 (d, *J* = 5.7 Hz, 2H), 4.38–4.30 (m, 2H), 4.22 (t, *J* = 7.0 Hz, 1H), 4.15–4.08 (m, 1H), 3.21–3.13 (m, 2H), 2.51 (s, 2H), 2.53–2.41 (m, 2H), 1.85 (mz, 1H), 1.74–1.64 (m, 1H), 1.55 (m, 2H), 1.43 (m, 2H), 1.32 (s, 3H). $^{13}$C-NMR (MeOD, 125 MHz) δ 176.51, 173.73, 172.44, 158.81, 145.52, 145.35, 142.74, 133.78, 128.94, 128.33, 128.31, 126.45, 126.42, 121.06, 118.61, 71.44, 68.08, 66.32, 55.65, 48.57, 47.26, 46.79, 40.13, 32.64, 30.02, 27.76,24.46. HRMS (ESI) calculated *m*/*z* for $[M+Na]^+$: 553.2550 found: 553.2549.

**H3K9hmg peptide synthesis and purification.**

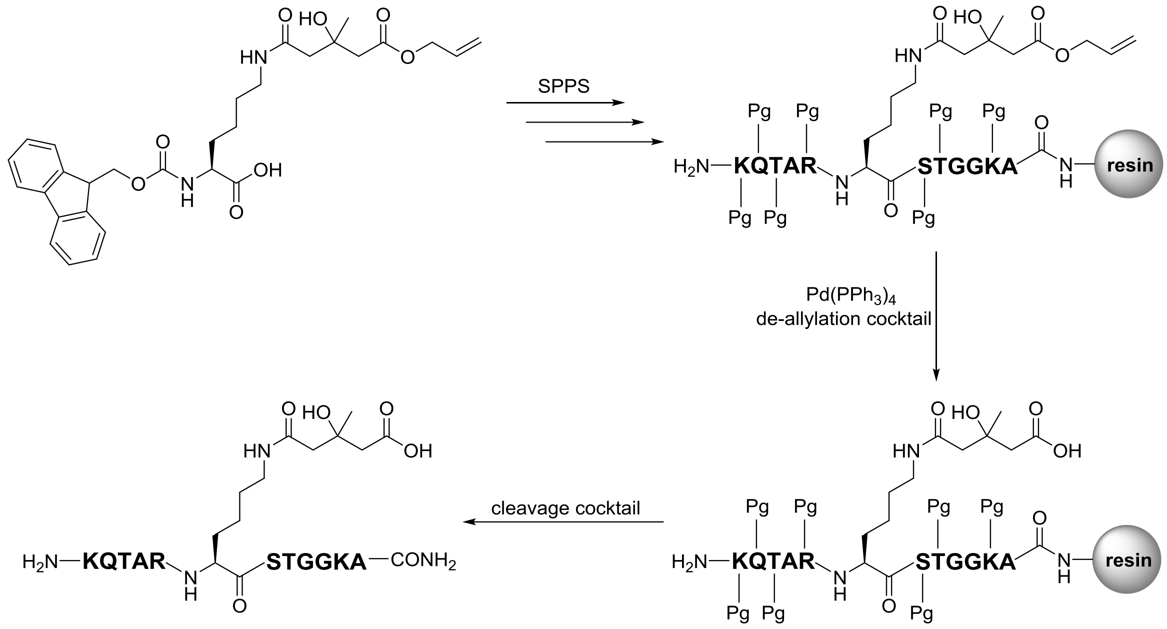


**Scheme S3.** Synthetic route of H3K9hmg peptide. SPPS = solid-phase peptide synthesis, Pg = acid labile protecting group, de-allylation cocktail: 2.5% *N*-methyl morpholine, 5% acetic acid in dry dichloromethane (v/v), cleavage cocktail: 2.5% triisopropylsilane, 1.5% $H_2O$ and, 1% thioanisole in trifluoroacetic acid (TFA) (v/v).

Peptide was synthesized on Rink-Amide MBHA resin followed standard Fmoc-based solid-phase peptide synthesis protocol. After the coupling of all amino acids, the last Fmoc group was removed, followed by the on-beads O-allylester deprotection. The resin was rinsed with 5 mL anhydrous dichloromethane for 3 times. The resin was charged in a flame-dried 10 mL round bottom flask with argon protection. To another 25 mL round bottom flask, 3.7 mL anhydrous dichloromethane was de-gassed with argon for 20 min and 100 μL *N*-methyl morpholine and 200 μL acetic acid were added to prepare the de-allylation cocktail. $Pd(PPh_3)_4$ (87 mg 0.075 mmol) was dissolved in the de-allylation cocktail and the resulting solution was subjected to the round bottom flask containing resin under argon protection. The reaction was kept stirring for 2 hours. The solution was discarded by

filtration and the beads were washed with 5% DIEA solution (v/v in dry DMF) and followed by washing with 5 mL dichloromethane for 5 times. The deprotection procedure should be repeated for another time to ensure a full removal of the allyl ester. The resulting resin was readily subjected to cleavage with cleavage cocktail containing 95% trifluoroacetic acid, 2.5% triisopropylsilane, 1.5% $H_2O$ and, 1% thioanisole for 2 h. Peptides were purified by preparative HPLC with an XBridge Prep OBDTM C18 column (30 mm × 250 mm, 10 μm, Waters). Mobile phase used were water with 0.1% TFA (buffer A) and 90% ACN in water with 0.1% TFA (buffer B). The purity (> 95%) and identity of peptides were confirmed by LC-MS.

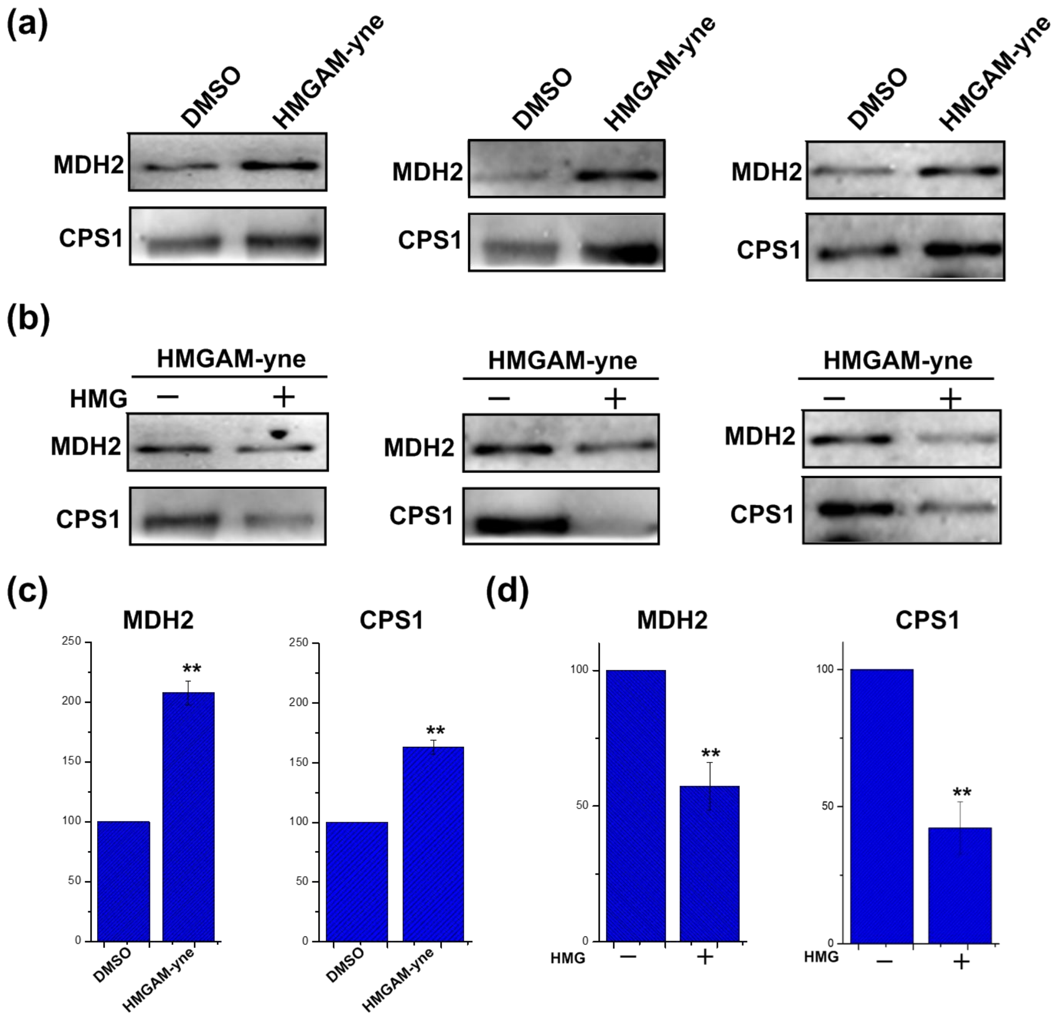


**Figure S1. Metabolic labeling of MDH2 and CPS1 by HMGAM-yne.** (**a**) Immunoblotting analyses showing the enrichment of two known protein substrates of HMGylation, CPS1 and MDH2, by HMGAM-yne. (**b**) Immunoblotting analyses showing the metabolic labelling of MDH2 and CPS1 with HMGAM-yne in the absence or presence of HMG as a competitor. After metabolic labeling, the labeled proteins were then conjugated were conjugated to biotin and isolated by high-capacity streptavidin beads, followed by immunoblotting analysis. (**c**) Quantitative analysis of immunoblotting results (**a-b**). Error bars indicated ± s.e. three independent biological replicates. The p values are based on the Student's t test. *p<0.05, **p <0.01, ***p <0.001.

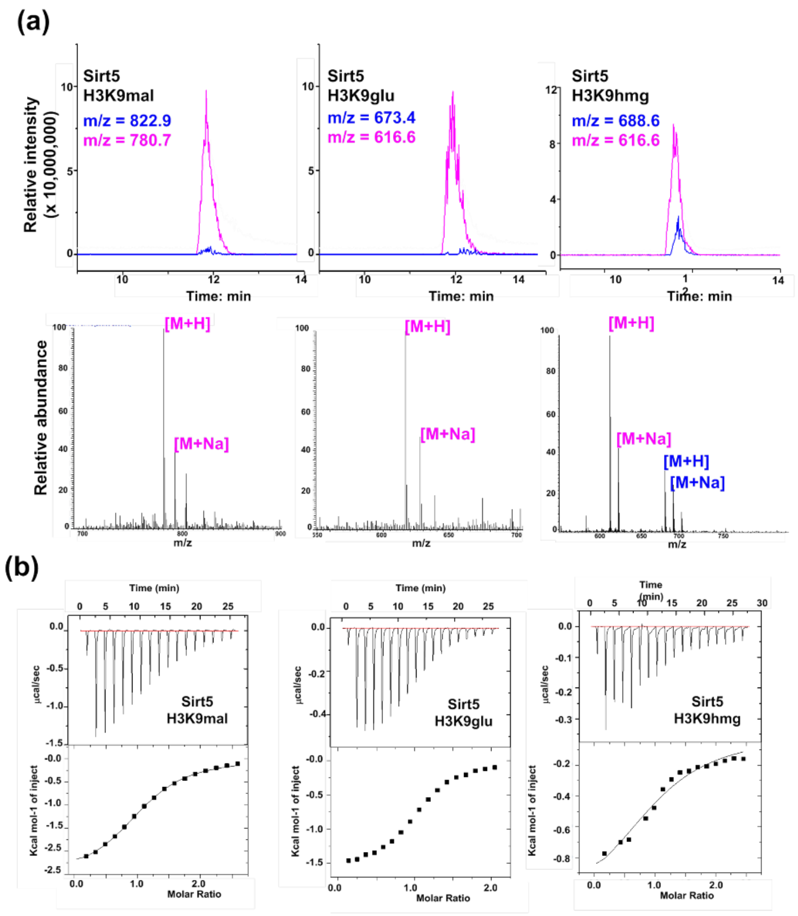


(c)

| Peptide | H3K9mal | H3K9glu | H3K9hmg |
|---|---|---|---|
| $K_d$ | 34.97 ± 1.6 µM | 12.55 ± 0.4 µM | 61.73 ± 1.8 µM |
| N | 1.07 ± 0.0998 Sites | 1.07 ± 0.0998 Sites | 1.07 ± 0.0998 Sites |
| ΔH | -2541 ± 32.95 cal/mol | -1579 ± 7.859 cal/mol | -1167 ± 162.9 cal/mol |
| ΔS | 11.9 cal/mol/deg | 17.1 cal/mol/deg | 15.3 cal/mol/deg |

**Figure S2. Sirt5 regulates the dynamics of lysine malonylation, glutarylation and HMGylation**. (**a**) The hydrolysis of H3K9mal/glu/hmg by Sirt5 was analyzed by liquid chromatography–mass spectrometry (LC-MS). Pink traces show ion intensity for the masses of unmodified peptide; and blue traces show ion intensity for the masses of malonylted/glutarylated/HMGylated peptide. (**b**) Isothermal titration calorimetry (ITC) measurement for the binding affinity of Sirt5 towards the H3K9mal/glu/hmg peptides. (**c**) A summary of the dissociation constants ($K_d$), enthalpy changes ($\Delta H$), and andentropy changes ($\Delta S$) of Sirt5 binding to H3K9mal/glu/hmg peptides.

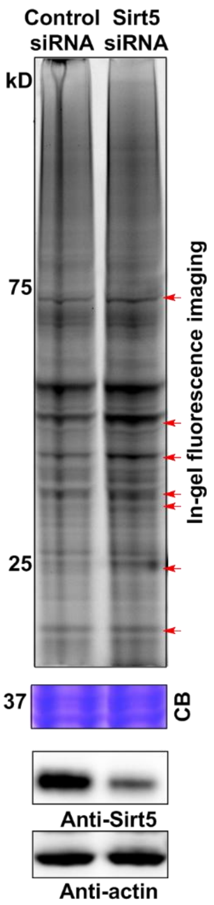


**Figure S3. Sirt5 regulates the dynamics of lysine HMGylation in living cell**. In-gel fluorescence analyses showing that Sirt5 knockdown caused the accumulations of lysine HMGylation at some protein bands indicated with red arrow. Coomassie-blue (CB) staining showing the equal loading. Immunoblotting analysis showing Sirt5 knockdown efficiency. γ-actin was used as a loading control.

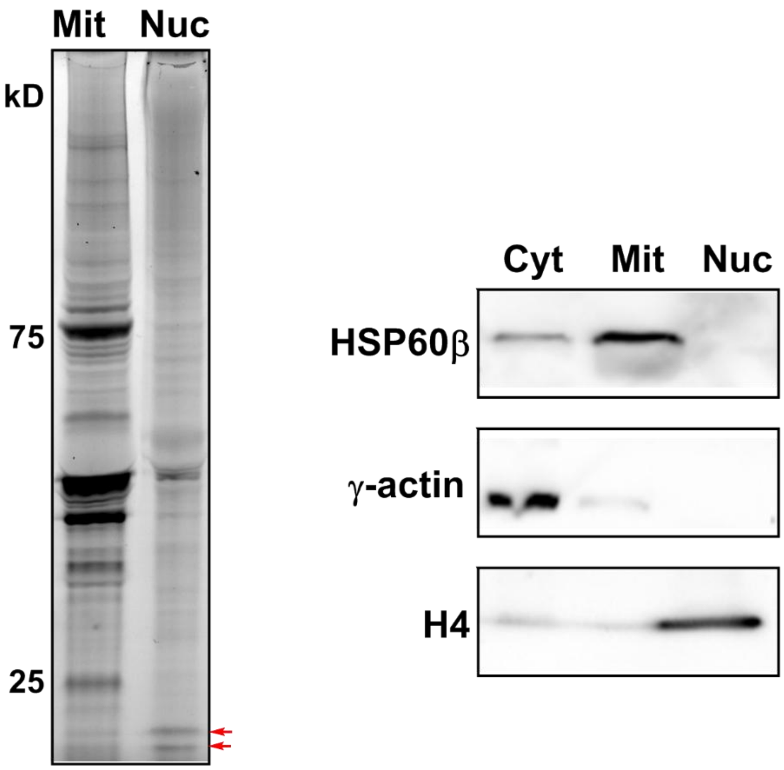


**Figure S4. Metabolic labeling of mitochondrial (Mit) and nuclear (Nuc) proteins by HMGAM-yne.** Nuclear extracted proteins with molecular weight around 15 kD was marked by red arrows. Immunoblotting analysis shows the purity of the mitochondrial extraction.

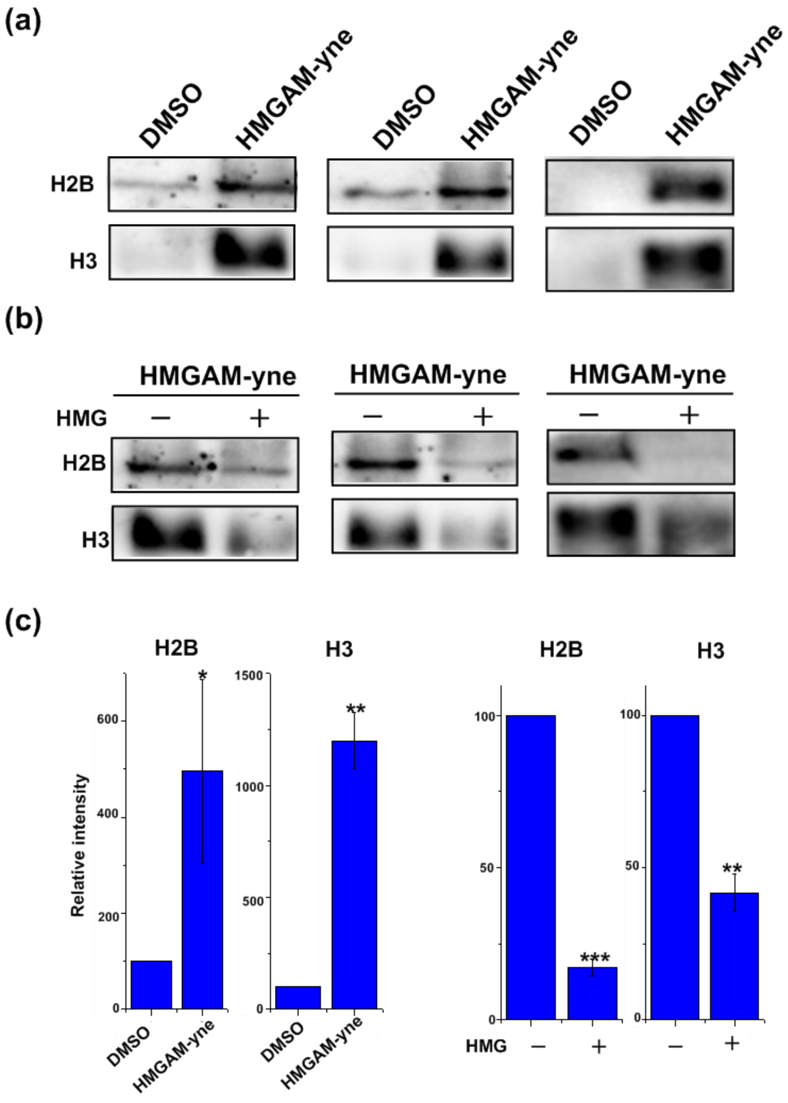


**Figure S5. Metabolic labeling of H2B and H3 by HMGAM-yne.** (**a**) Immunoblotting analyses showing the enrichment histone H2B and H3 by HMGAM-yne. (**b**) Immunoblotting analyses showing the metabolic labelling of H2B and H3 with HMGAM-yne in the absence or presence of HMG as a competitor. After metabolic labeling, the labeled proteins were conjugated to biotin and isolated by high-capacity streptavidin beads, followed by immunoblotting analysis. (**c**) Quantitative analysis of immunoblotting results (**a-b**). Error bars indicated ± s.e. three independent biological replicates. The p values are based on the Student's t test. *p<0.05, **p <0.01, ***p <0.001.

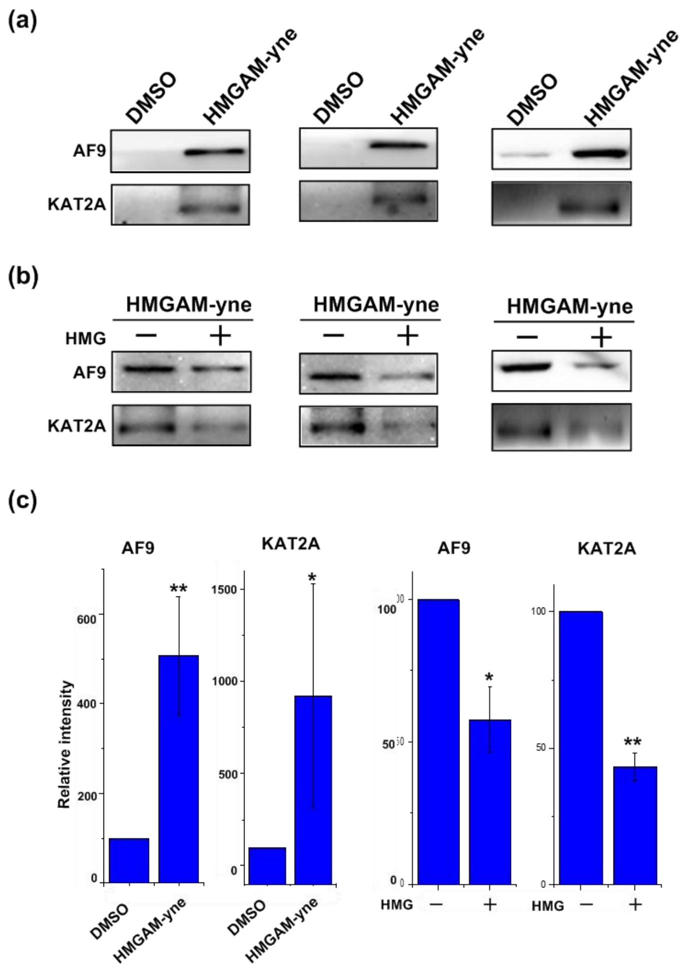


**Figure S6. Metabolic labeling of AF9 and KAT2A by HMGAM-yne.** (**a**) Immunoblotting analyses showing the enrichment of two nuclear proteins, AF9 and KAT2A, by HMGAM-yne. (**b**) Immunoblotting analyses showing the metabolic labelling of AF9 and KAT2A with HMGAM-yne in the absence or presence of HMG as a competitor. After metabolic labeling, the labeled proteins were conjugated to biotin and isolated by high-capacity streptavidin beads, followed by immunoblotting analysis. (**c**) Quantitative analysis of immunoblotting results (**a-b**). Error bars indicated ±s.e. from three independent biological replicates. The p values are based on the Student's t test. *p<0.05, **p <0.01, ***p <0.001.

**Table S1. The estimated incorporation rates of HMGylated lysine in histones.**

| No. | Protein | Position | Sequence | Incorporation rate % |
|---|---|---|---|---|
| ***1*** | Histone H4 | 59 | $^{56}$ $_{pr}$VLK$_{hmg}$VFLENVIR$^{67}$ | 1.95 |
| ***2*** | Histone H3 | 27 | $^{27}$ $_{pr}$K$_{hmg}$SAPATGGVK$^{36}$ | 4.8 |
| ***3*** | Histone H3 | 18 | $^{18}$K$_{hmg}$QLATK$_{pr}$AAR$^{26}$ | <0.1 |
| ***4*** | Histone H3 | 9 | $^{9}$K$_{hmg}$STGGK$_{hmg}$APR$^{17}$ | 3 |
| ***4*** | Histone H3 | 14 | $^{9}$K$_{hmg}$STGGK$_{hmg}$APR$^{17}$ | 3 |
| ***5*** | Histone H2A | 119 | $^{119}$ $_{pr}$K$_{hmg}$TESHHK$^{125}$ | 5 |

**Note S1**. **Characterization of peptides.**

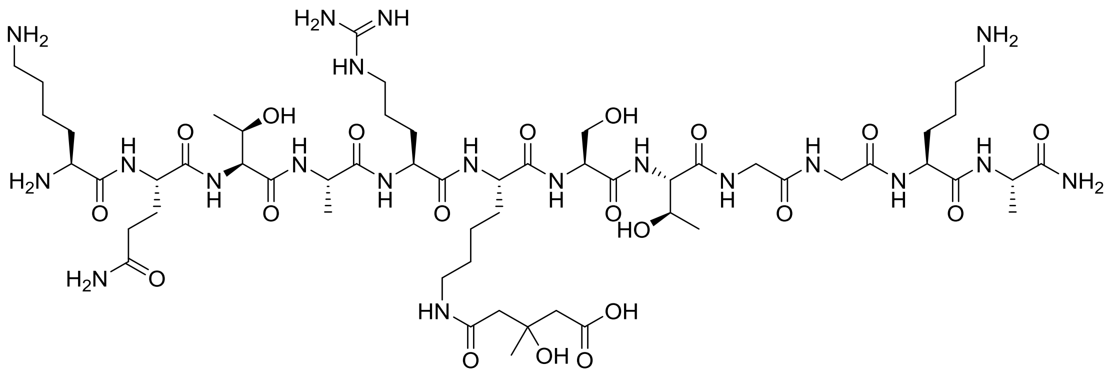

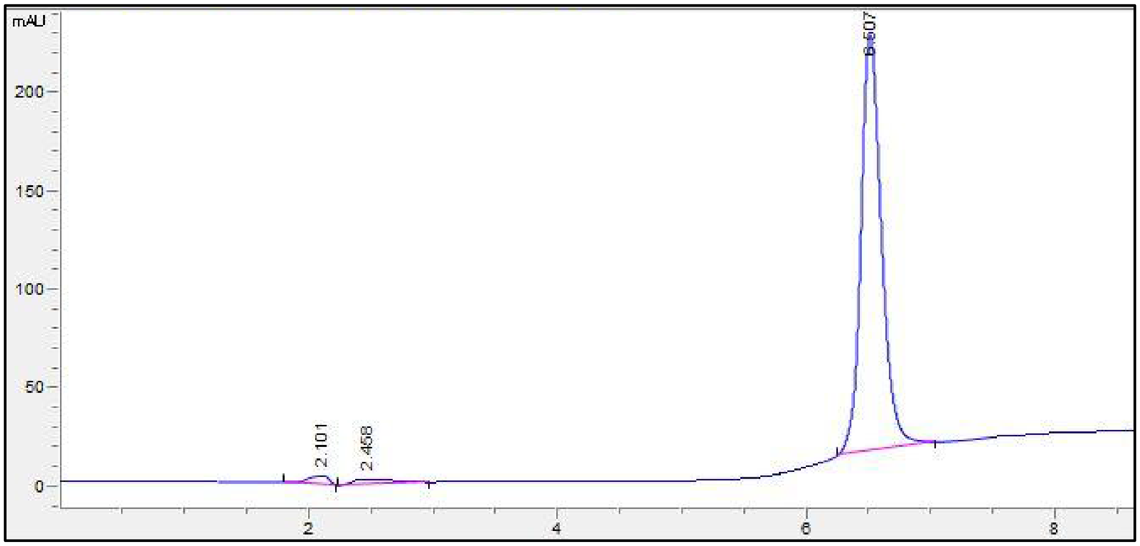

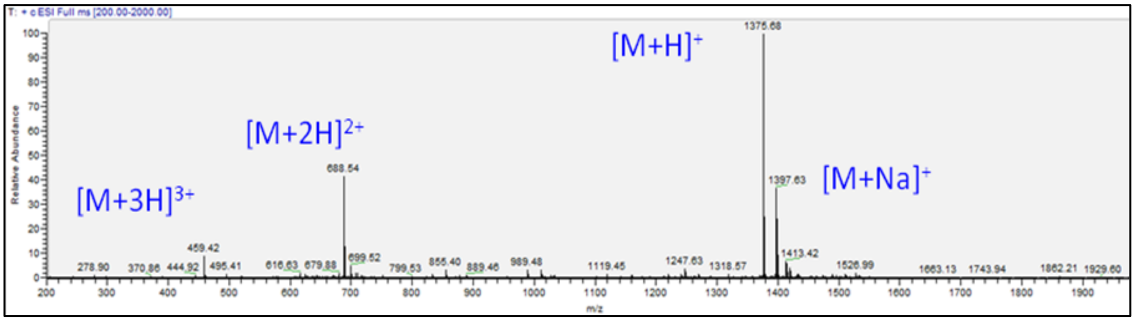


LC–MS analysis of H3K9hmg peptide: Calculated *m/z* for $[M + H]^{+}$: 1375.77, found: 1375.68; calculated *m/z* for $[M + 2H]^{2+}$: 688.39, found: 688.54; calculated *m/z* for $[M + 3H]^{3+}$: 459.26, found: 459.42.

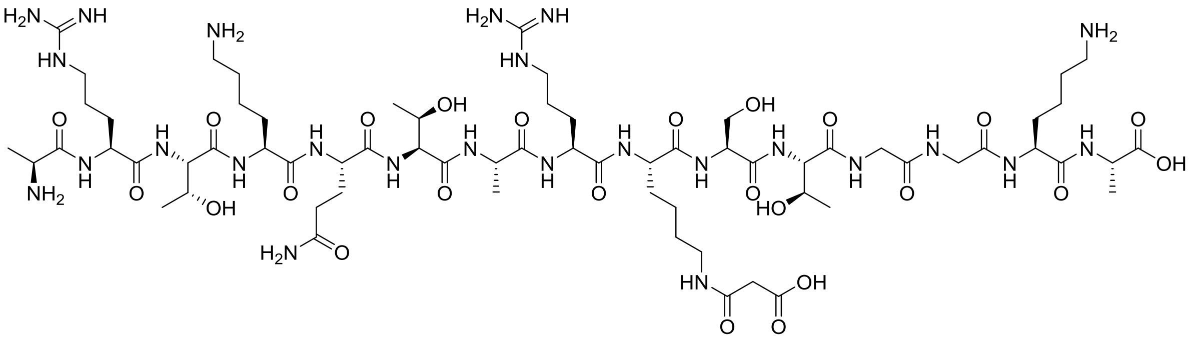

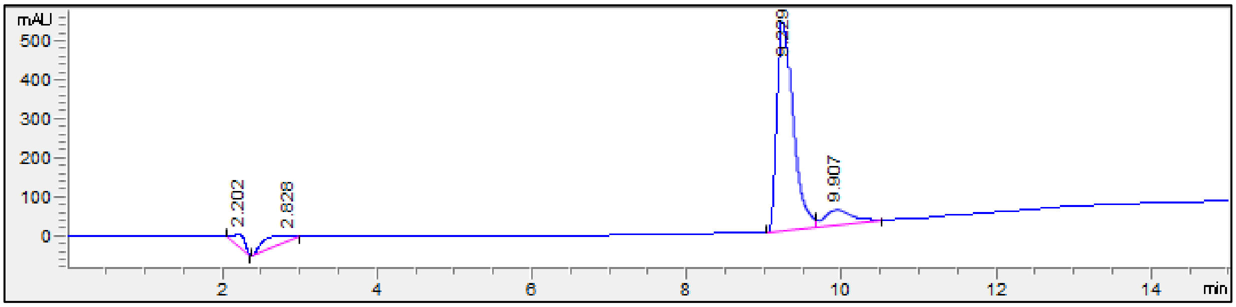


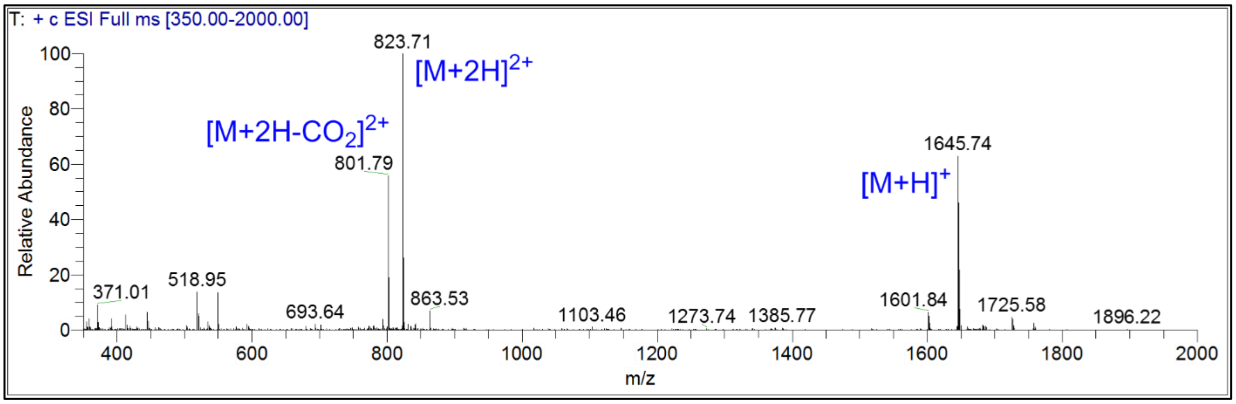


LC–MS analysis of H3K9mal (1-15) peptide: Calculated *m/z* for $[M + H]^{+}$: 1645.9, found: 1645.74; calculated *m/z* for $[M + 2H]^{2+}$: 823.45, found: 823.71; calculated *m/z* for $[M + 2H\text{-}CO_2]^{2+}$: 801.71, found: 801.79.

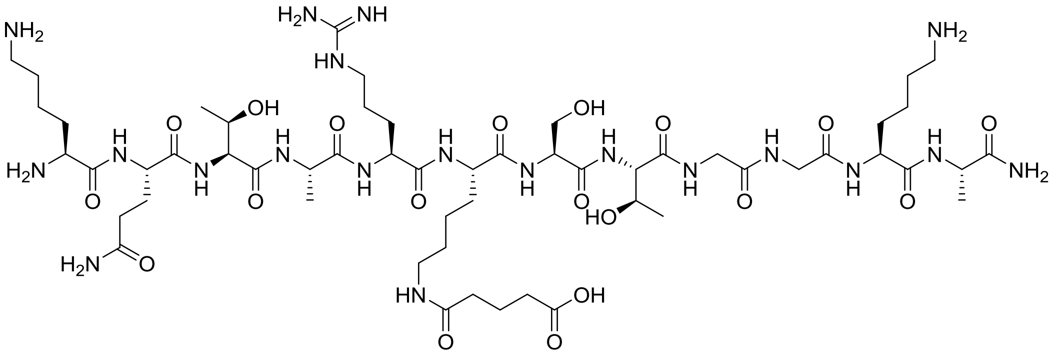

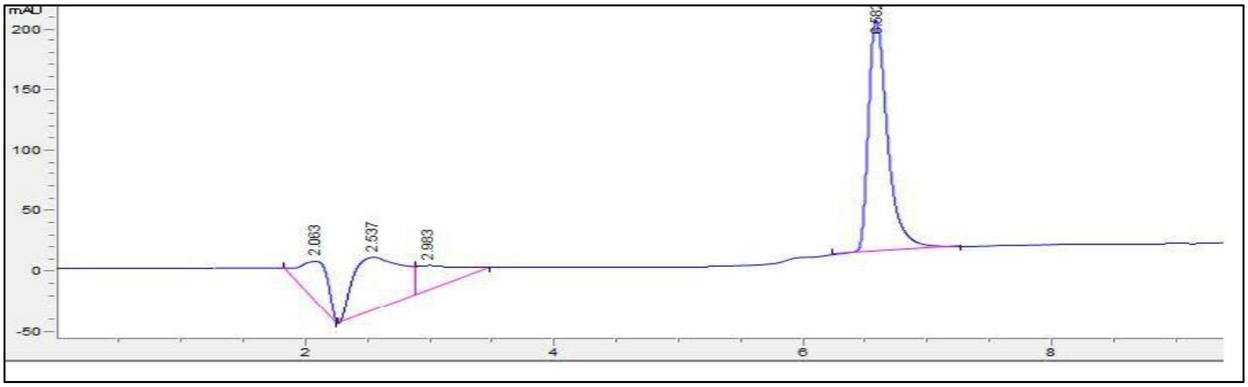

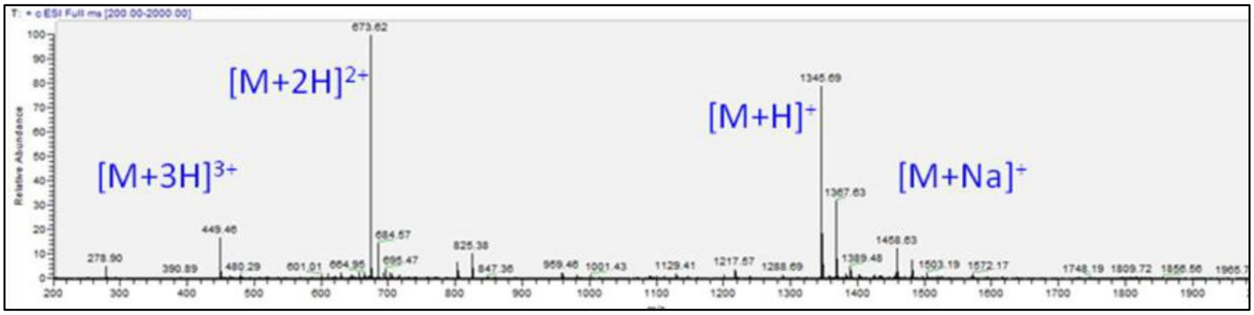


LC–MS analysis of H3K9glu peptide: Calculated *m/z* for $[M + H]^+$: 1345.76, found: 1345.69; calculated *m/z* for $[M + 2H]^{2+}$: 673.38, found: 673.62; calculated *m/z* for $[M + 3H]^{3+}$: 449.25, found: 449.46.

**Note S2. MS/MS spectra of lysine HMGylated peptides in histones.**

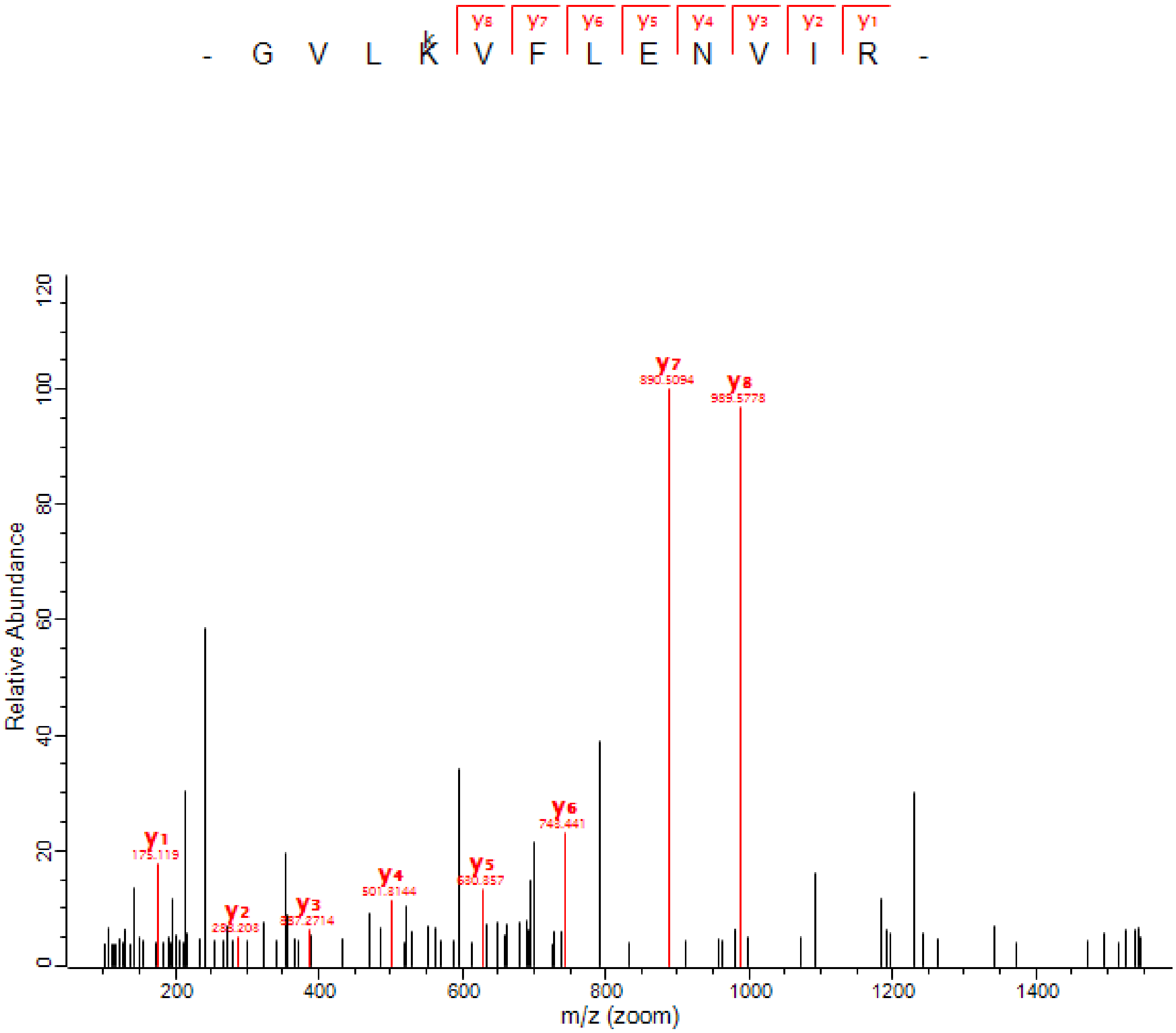


The CID MS/MS spectrum of a doubly charged tryptic-digest peptide, $_{pr}{}^{56}$GVLK$_{hmg}$VFLENVIR$^{67}$, from histone H4.

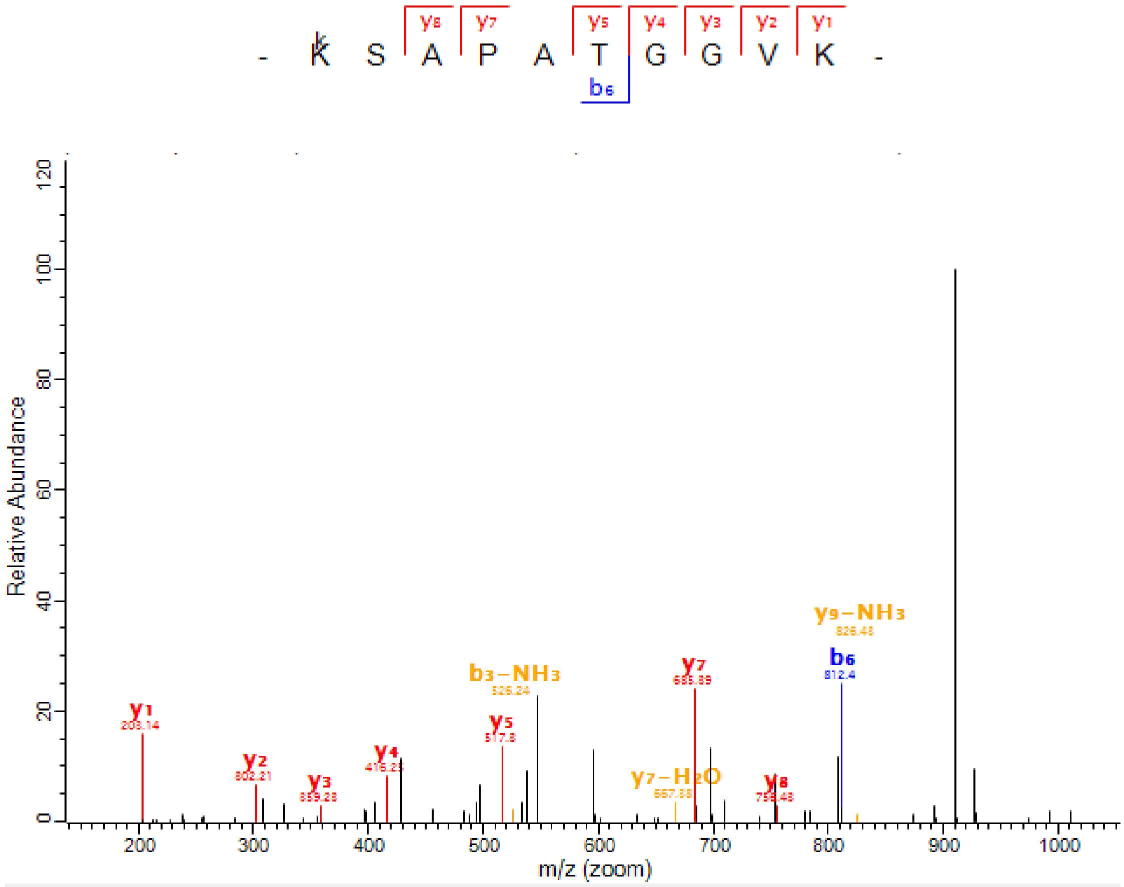


The CID MS/MS spectrum of a doubly charged tryptic-digest peptide, $_{pr}{}^{27}K_{hmg}SAPATGGVK^{36}$, from histone H3.

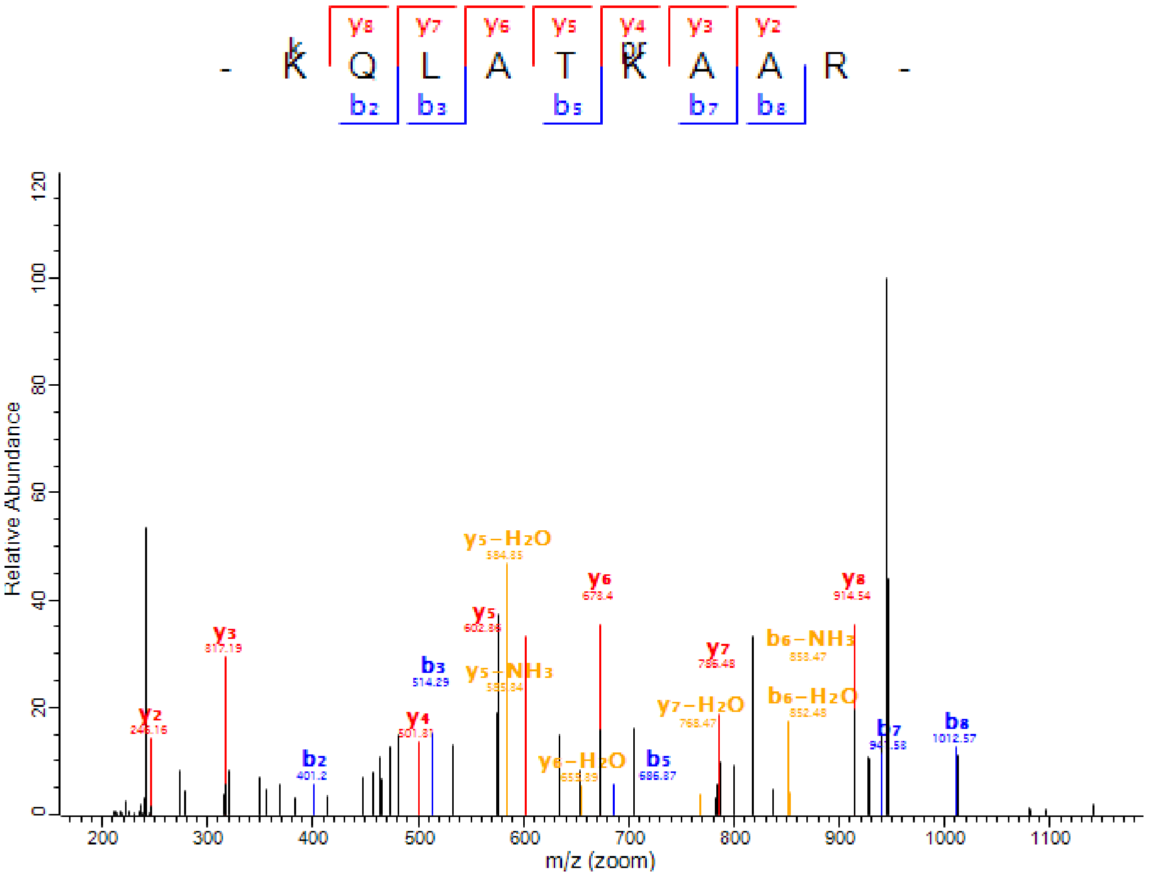


The CID MS/MS spectrum of a doubly charged tryptic-digest peptide, $^{18}K_{hmg}QLATK_{pr}AAR^{26}$, from histone H3.

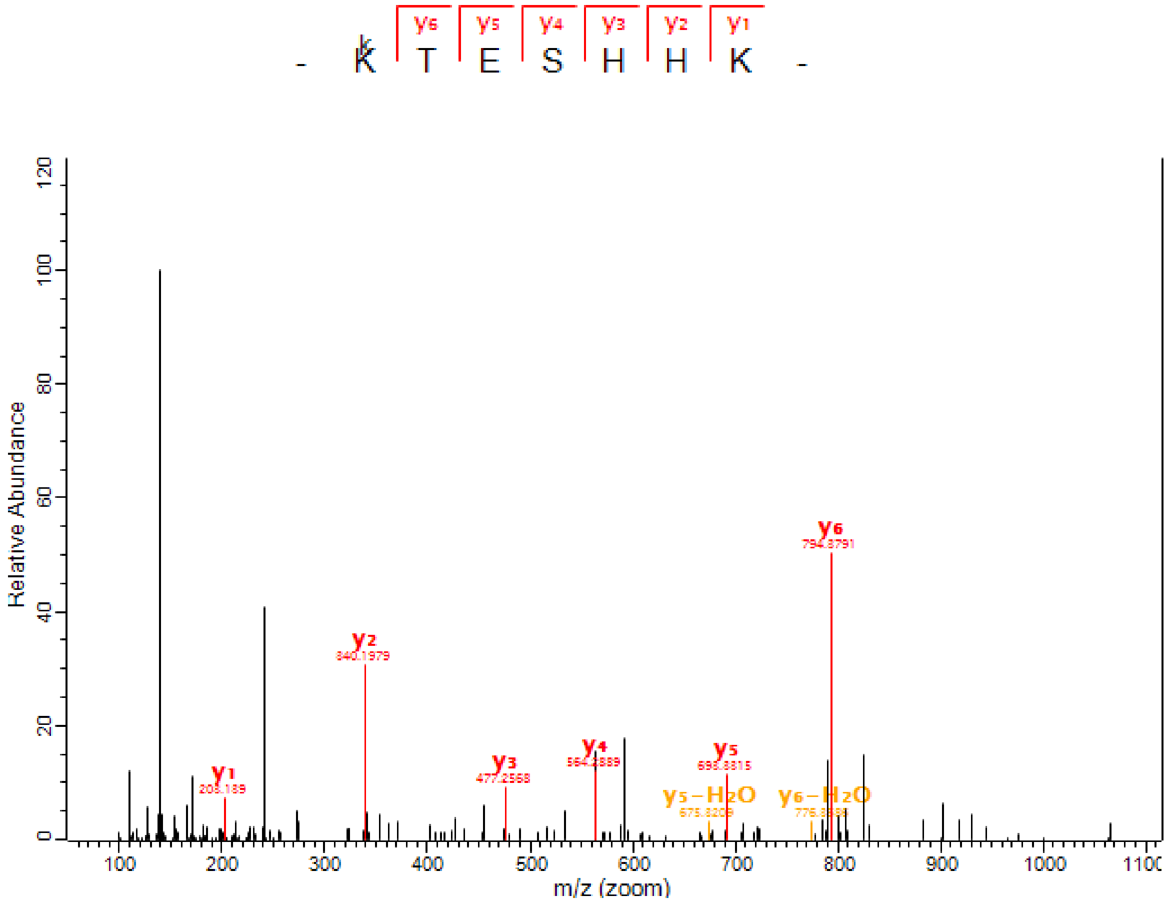


The CID MS/MS spectrum of a doubly charged tryptic-digest peptide, $_{pr}{}^{119}K_{hmg}TESHHK^{125}$, from histone H2A.

**Note S3. $^{1}$H- and $^{13}$C-NMR spectra for all the compounds synthesized in this study**

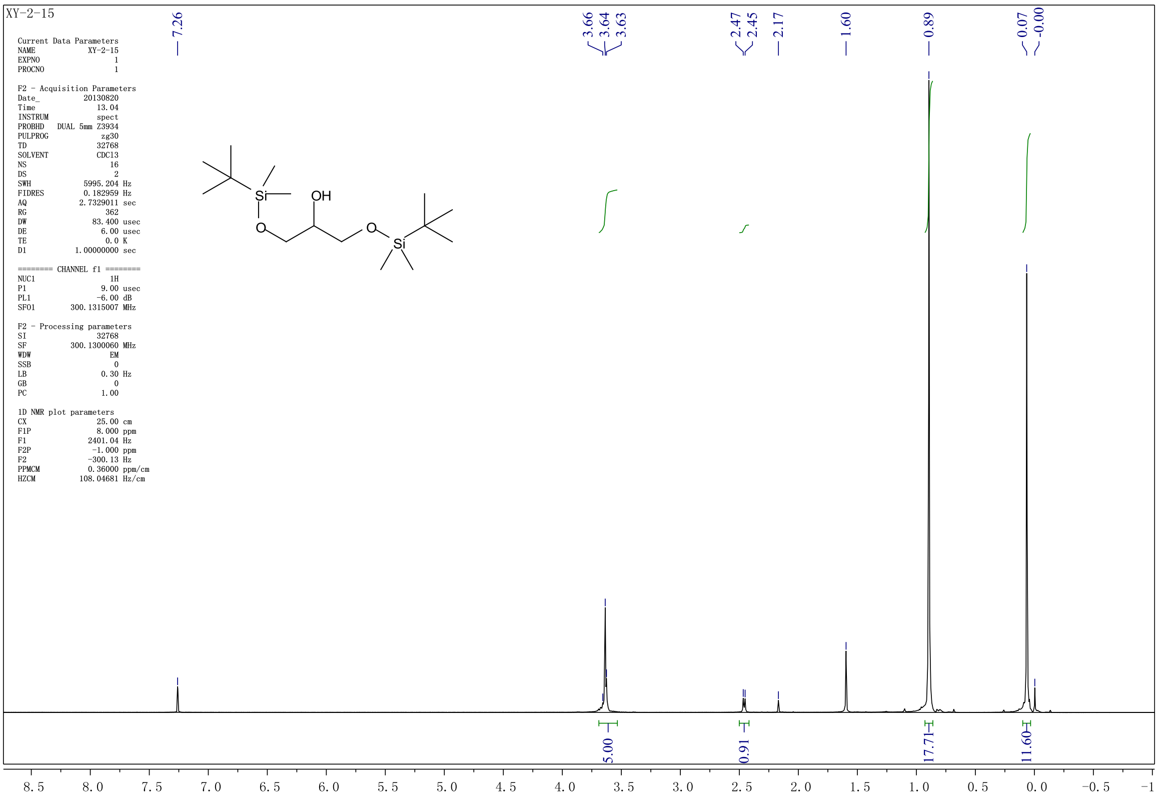
XY-2-15
Current Data Parameters
NAME XY-2-15
EXPNO 1
PROCNO 1
F2 - Acquisition Parameters
Date_ 20130820
Time 13.04
INSTRUM spect
PROBHD DUAL 5mm Z3934
PULPROG zg30
TD 32768
SOLVENT CDCl3
NS 16
DS 2
SWH 5995.204 Hz
FIDRES 0.182959 Hz
AQ 2.7329011 sec
RG 362
DW 83.400 usec
DE 6.00 usec
TE 0.0 K
D1 1.00000000 sec
======== CHANNEL f1 ========
NUC1 1H
P1 9.00 usec
PL1 -6.00 dB
SFO1 300.1315007 MHz
F2 - Processing parameters
SI 32768
SF 300.1300060 MHz
WDW EM
SSB 0
LB 0.30 Hz
GB 0
PC 1.00
1D NMR plot parameters
CX 25.00 cm
F1P 8.000 ppm
F1 2401.04 Hz
F2P -1.000 ppm
F2 -300.13 Hz
PPMCM 0.36000 ppm/cm
HZCM 108.04681 Hz/cm
Si
OH
O
O
Si
7.26
3.66
3.64
3.63
2.47
2.45
2.17
1.60
0.89
0.07
-0.00
5.00
0.91
17.71
11.60
8.5 8.0 7.5 7.0 6.5 6.0 5.5 5.0 4.5 4.0 3.5 3.0 2.5 2.0 1.5 1.0 0.5 0.0 -0.5 -1

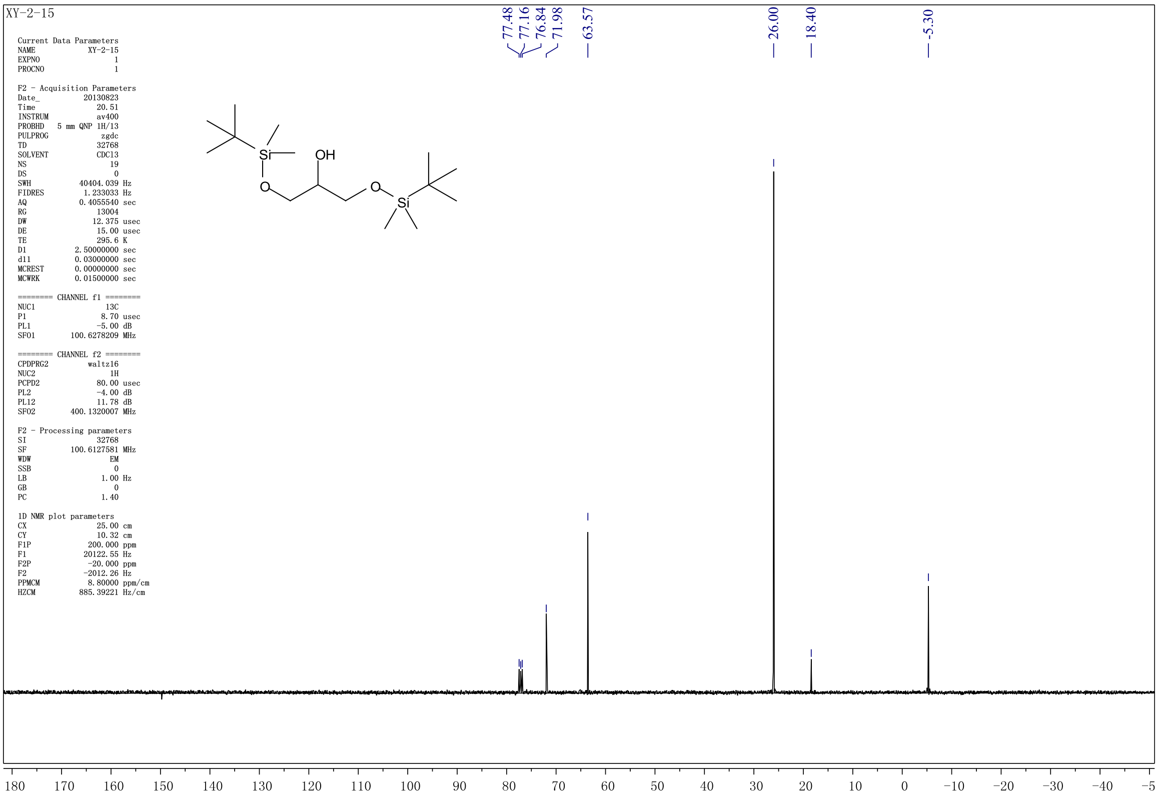
XY-2-15
Current Data Parameters
NAME XY-2-15
EXPNO 1
PROCNO 1
F2 - Acquisition Parameters
Date_ 20130823
Time 20.51
INSTRUM av400
PROBHD 5 mm QNP 1H/13
PULPROG zgdc
TD 32768
SOLVENT CDCl3
NS 19
DS 0
SWH 40404.039 Hz
FIDRES 1.233033 Hz
AQ 0.4055540 sec
RG 13004
DW 12.375 usec
DE 15.00 usec
TE 295.6 K
D1 2.50000000 sec
d11 0.03000000 sec
MCREST 0.00000000 sec
MCWRK 0.01500000 sec
======== CHANNEL f1 ========
NUC1 13C
P1 8.70 usec
PL1 -5.00 dB
SFO1 100.6278209 MHz
======== CHANNEL f2 ========
CPDPRG2 waltz16
NUC2 1H
PCPD2 80.00 usec
PL2 -4.00 dB
PL12 11.78 dB
SFO2 400.1320007 MHz
F2 - Processing parameters
SI 32768
SF 100.6127581 MHz
WDW EM
SSB 0
LB 1.00 Hz
GB 0
PC 1.40
1D NMR plot parameters
CX 25.00 cm
CY 10.32 cm
F1P 200.000 ppm
F1 20122.55 Hz
F2P -20.000 ppm
F2 -2012.26 Hz
PPMCM 8.80000 ppm/cm
HZCM 885.39221 Hz/cm
Si
OH
O
O
Si
77.48
77.16
76.84
71.98
63.57
26.00
18.40
-5.30
180 170 160 150 140 130 120 110 100 90 80 70 60 50 40 30 20 10 0 -10 -20 -30 -40 -5

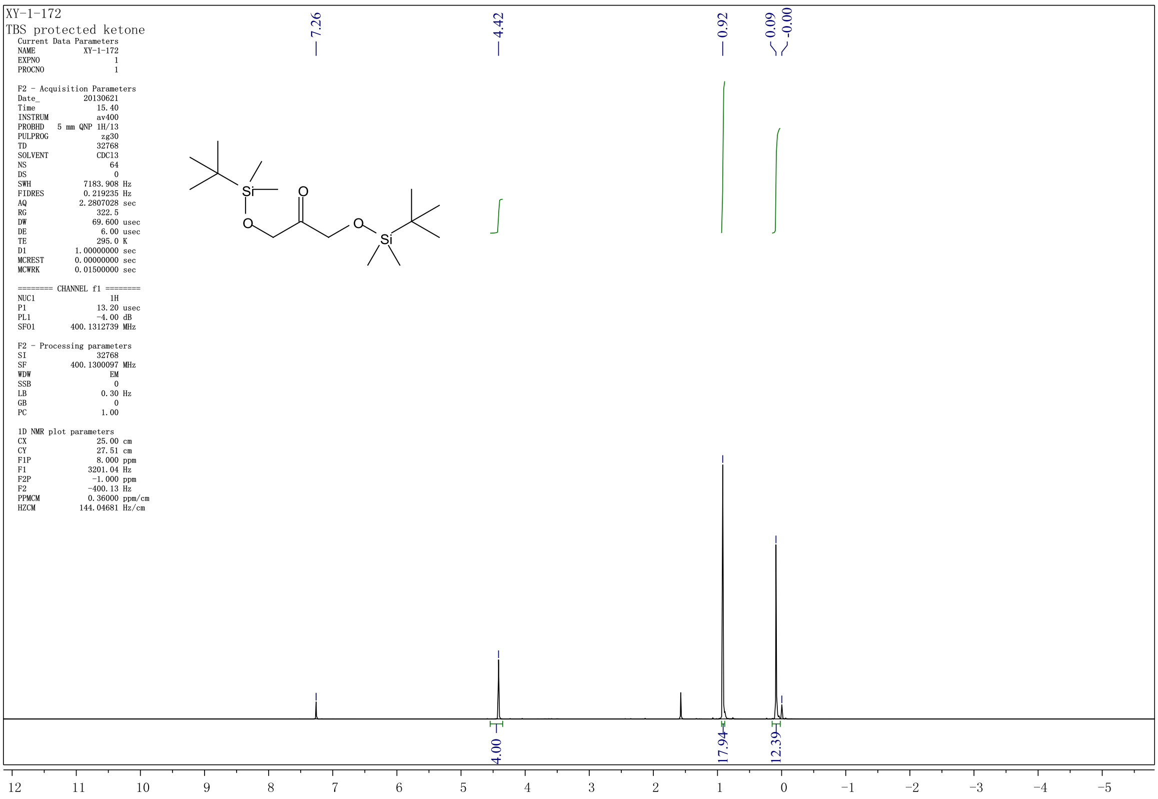
XY-1-172
TBS protected ketone
Current Data Parameters
NAME XY-1-172
EXPNO 1
PROCNO 1
F2 - Acquisition Parameters
Date_ 20130621
Time 15.40
INSTRUM av400
PROBHD 5 mm QNP 1H/13
PULPROG zg30
TD 32768
SOLVENT CDCl3
NS 64
DS 0
SWH 7183.908 Hz
FIDRES 0.219235 Hz
AQ 2.2807028 sec
RG 322.5
DW 69.600 usec
DE 6.00 usec
TE 295.0 K
D1 1.00000000 sec
MCREST 0.00000000 sec
MCWRK 0.01500000 sec
======== CHANNEL f1 ========
NUC1 1H
P1 13.20 usec
PL1 -4.00 dB
SFO1 400.1312739 MHz
F2 - Processing parameters
SI 32768
SF 400.1300097 MHz
WDW EM
SSB 0
LB 0.30 Hz
GB 0
PC 1.00
1D NMR plot parameters
CX 25.00 cm
CY 27.51 cm
F1P 8.000 ppm
F1 3201.04 Hz
F2P -1.000 ppm
F2 -400.13 Hz
PPMCM 0.36000 ppm/cm
HZCM 144.04681 Hz/cm
7.26
4.42
0.92
0.09
-0.00
4.00
17.94
12.39
12 11 10 9 8 7 6 5 4 3 2 1 0 −1 −2 −3 −4 −5

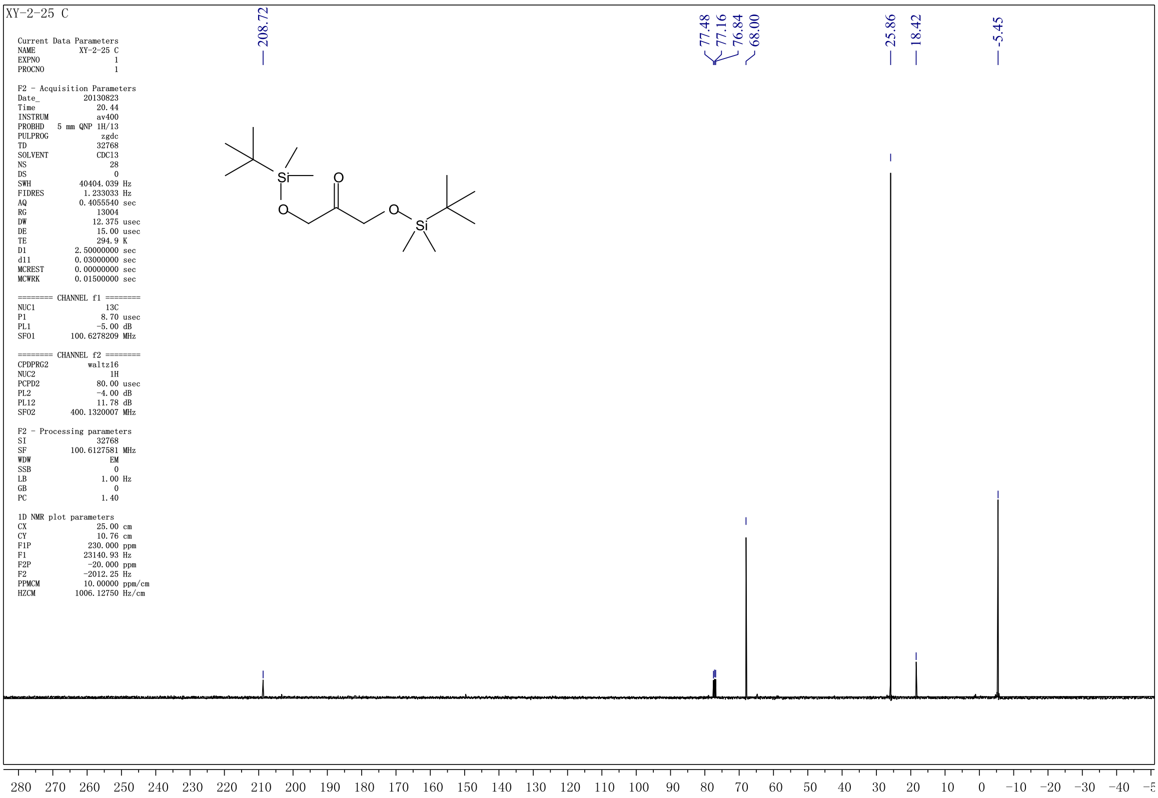
XY-2-25 C
Current Data Parameters
NAME XY-2-25 C
EXPNO 1
PROCNO 1
F2 - Acquisition Parameters
Date_ 20130823
Time 20.44
INSTRUM av400
PROBHD 5 mm QNP 1H/13
PULPROG zgdc
TD 32768
SOLVENT CDCl3
NS 28
DS 0
SWH 40404.039 Hz
FIDRES 1.233033 Hz
AQ 0.4055540 sec
RG 13004
DW 12.375 usec
DE 15.00 usec
TE 294.9 K
D1 2.50000000 sec
d11 0.03000000 sec
MCREST 0.00000000 sec
MCWRK 0.01500000 sec
======== CHANNEL f1 ========
NUC1 13C
P1 8.70 usec
PL1 -5.00 dB
SFO1 100.6278209 MHz
======== CHANNEL f2 ========
CPDPRG2 waltz16
NUC2 1H
PCPD2 80.00 usec
PL2 -4.00 dB
PL12 11.78 dB
SFO2 400.1320007 MHz
F2 - Processing parameters
SI 32768
SF 100.6127581 MHz
WDW EM
SSB 0
LB 1.00 Hz
GB 0
PC 1.40
1D NMR plot parameters
CX 25.00 cm
CY 10.76 cm
F1P 230.000 ppm
F1 23140.93 Hz
F2P -20.000 ppm
F2 -2012.25 Hz
PPMCM 10.00000 ppm/cm
HZCM 1006.12750 Hz/cm
208.72
77.48
77.16
76.84
68.00
25.86
18.42
-5.45
280 270 260 250 240 230 220 210 200 190 180 170 160 150 140 130 120 110 100 90 80 70 60 50 40 30 20 10 0 −10 −20 −30 −40

XY-2-29

```
Current Data Parameters
NAME            XY-2-29
EXPNO                 1
PROCNO                1

F2 - Acquisition Parameters
Date_          20130828
Time              22.31
INSTRUM           av400
PROBHD   5 mm QNP 1H/13
PULPROG            zg30
TD                32768
SOLVENT           CDCl3
NS                   22
DS                    0
SWH            7183.908 Hz
FIDRES         0.219235 Hz
AQ            2.2807028 sec
RG                 35.9
DW               69.600 usec
DE                 6.00 usec
TE                298.0 K
D1           1.00000000 sec
MCREST       0.00000000 sec
MCWRK        0.01500000 sec

======== CHANNEL f1 ========
NUC1                 1H
P1                13.20 usec
PL1               -4.00 dB
SFO1        400.1312739 MHz

F2 - Processing parameters
SI                32768
SF          400.1300095 MHz
WDW                  EM
SSB                   0
LB                 0.30 Hz
GB                    0
PC                 1.00

1D NMR plot parameters
CX                25.00 cm
CY                49.51 cm
F1P               8.000 ppm
F1              3201.04 Hz
F2P              -1.000 ppm
F2              -400.13 Hz
PPMCM           0.36000 ppm/cm
HZCM          144.04681 Hz/cm
```

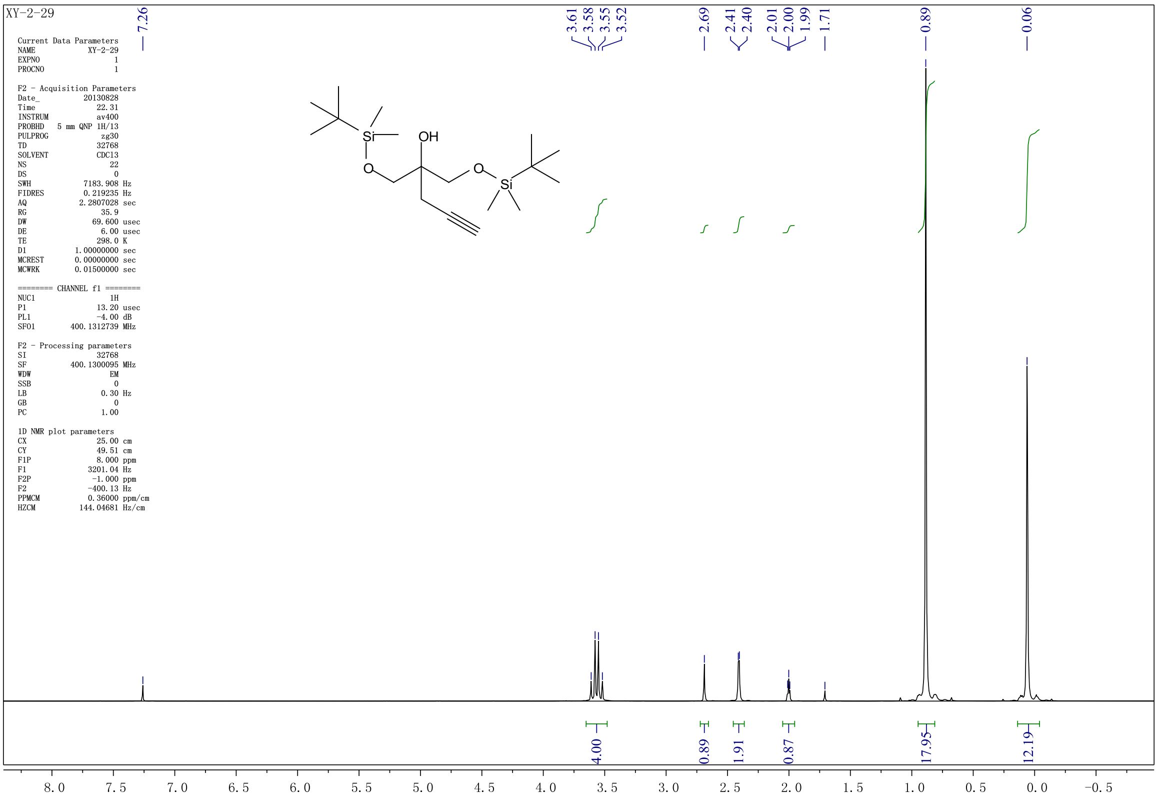


XY-2-29 C

```
Current Data Parameters
NAME          XY-2-29 C
EXPNO                 1
PROCNO                1

F2 - Acquisition Parameters
Date_          20130828
Time              22.35
INSTRUM           av400
PROBHD   5 mm QNP 1H/13
PULPROG            zgdc
TD                32768
SOLVENT           CDCl3
NS                   35
DS                    0
SWH           40404.039 Hz
FIDRES         1.233033 Hz
AQ            0.4055540 sec
RG                13004
DW               12.375 usec
DE                15.00 usec
TE                298.0 K
D1           2.50000000 sec
d11          0.03000000 sec
MCREST       0.00000000 sec
MCWRK        0.01500000 sec

======== CHANNEL f1 ========
NUC1                13C
P1                 8.70 usec
PL1               -5.00 dB
SFO1        100.6278209 MHz

======== CHANNEL f2 ========
CPDPRG2         waltz16
NUC2                 1H
PCPD2             80.00 usec
PL2               -4.00 dB
PL12              11.78 dB
SFO2        400.1320007 MHz

F2 - Processing parameters
SI                32768
SF          100.6127581 MHz
WDW                  EM
SSB                   0
LB                 1.00 Hz
GB                    0
PC                 1.40

1D NMR plot parameters
CX                25.00 cm
CY                36.31 cm
F1P             100.000 ppm
F1             10061.27 Hz
F2P             -10.000 ppm
F2             -1006.13 Hz
PPMCM           4.40000 ppm/cm

HZCM          442.69611 Hz/cm
```

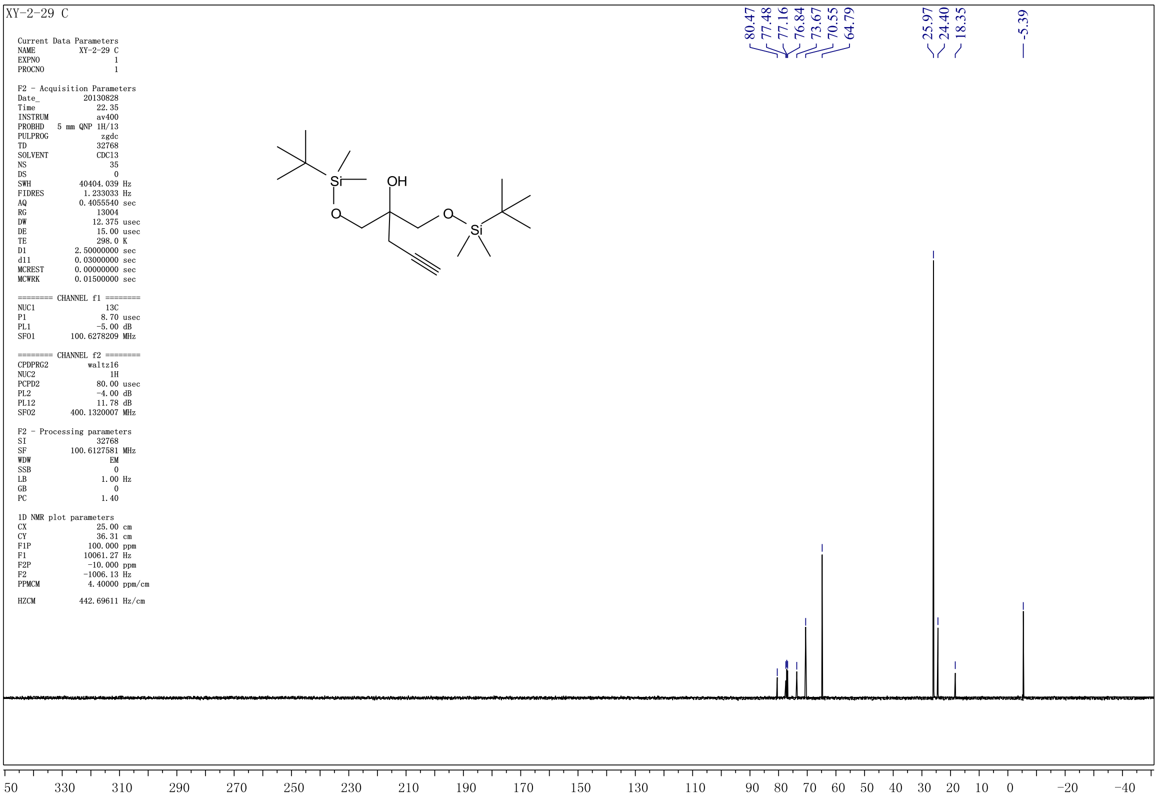

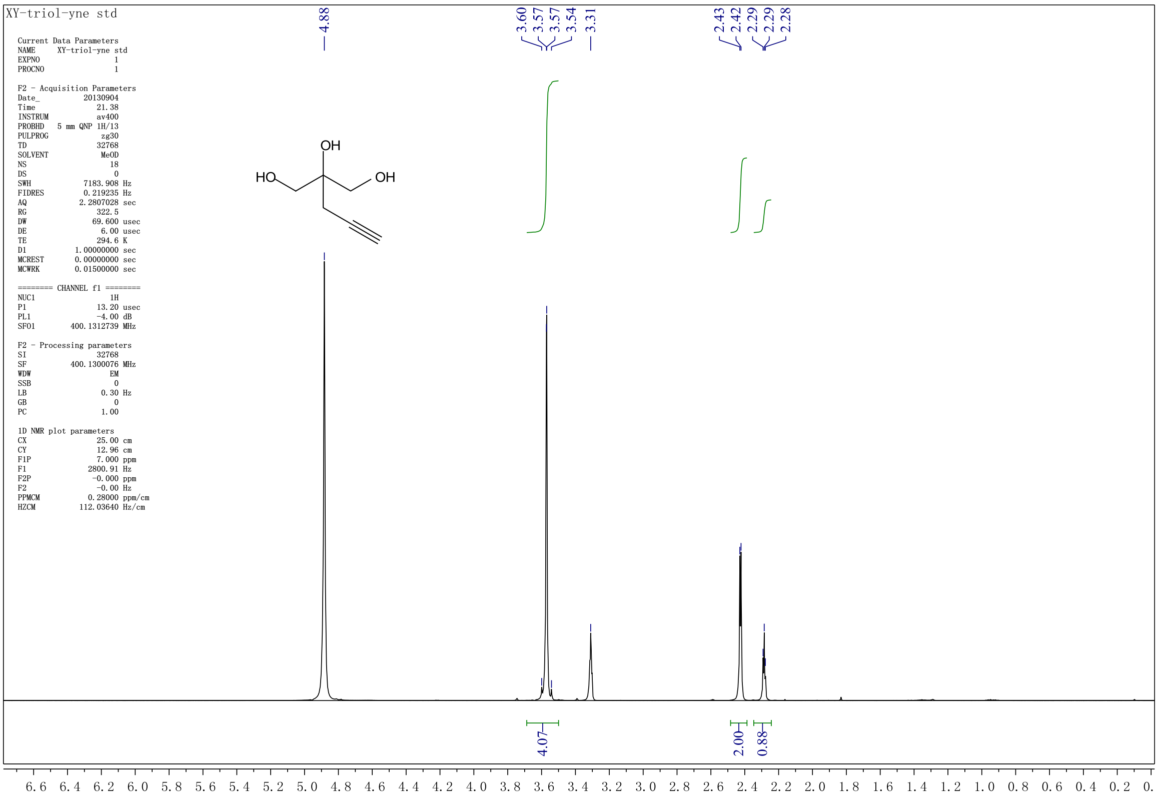
XY-triol-yne std
Current Data Parameters
NAME XY-triol-yne std
EXPNO 1
PROCNO 1
F2 - Acquisition Parameters
Date_ 20130904
Time 21.38
INSTRUM av400
PROBHD 5 mm QNP 1H/13
PULPROG zg30
TD 32768
SOLVENT MeOD
NS 18
DS 0
SWH 7183.908 Hz
FIDRES 0.219235 Hz
AQ 2.2807028 sec
RG 322.5
DW 69.600 usec
DE 6.00 usec
TE 294.6 K
D1 1.00000000 sec
MCREST 0.00000000 sec
MCWRK 0.01500000 sec
======== CHANNEL f1 ========
NUC1 1H
P1 13.20 usec
PL1 -4.00 dB
SFO1 400.1312739 MHz
F2 - Processing parameters
SI 32768
SF 400.1300076 MHz
WDW EM
SSB 0
LB 0.30 Hz
GB 0
PC 1.00
1D NMR plot parameters
CX 25.00 cm
CY 12.96 cm
F1P 7.000 ppm
F1 2800.91 Hz
F2P -0.000 ppm
F2 -0.00 Hz
PPMCM 0.28000 ppm/cm
HZCM 112.03640 Hz/cm
4.88
3.60
3.57
3.57
3.54
3.31
2.43
2.42
2.29
2.29
2.28
OH
HO
OH
4.07
2.00
0.88
6.6 6.4 6.2 6.0 5.8 5.6 5.4 5.2 5.0 4.8 4.6 4.4 4.2 4.0 3.8 3.6 3.4 3.2 3.0 2.8 2.6 2.4 2.2 2.0 1.8 1.6 1.4 1.2 1.0 0.8 0.6 0.4 0.2 0.

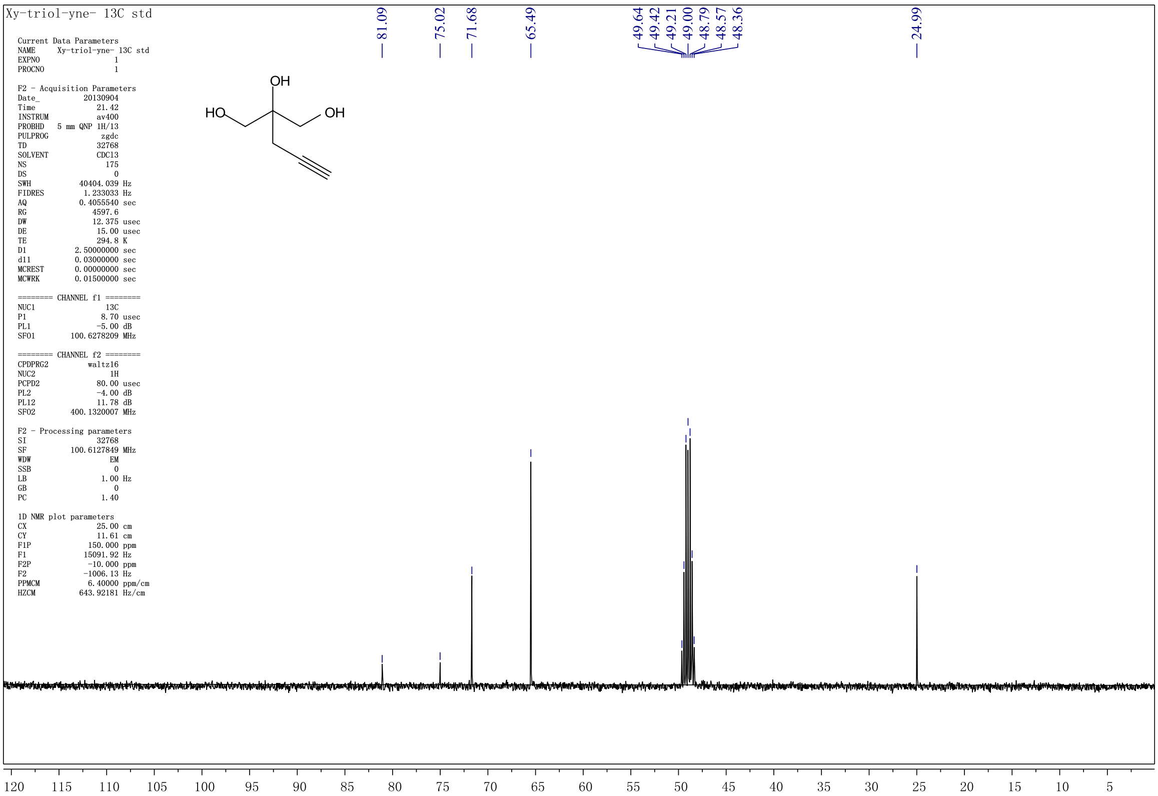
Xy-triol-yne- 13C std
Current Data Parameters
NAME Xy-triol-yne- 13C std
EXPNO 1
PROCNO 1
F2 - Acquisition Parameters
Date_ 20130904
Time 21.42
INSTRUM av400
PROBHD 5 mm QNP 1H/13
PULPROG zgdc
TD 32768
SOLVENT CDCl3
NS 175
DS 0
SWH 40404.039 Hz
FIDRES 1.233033 Hz
AQ 0.4055540 sec
RG 4597.6
DW 12.375 usec
DE 15.00 usec
TE 294.8 K
D1 2.50000000 sec
d11 0.03000000 sec
MCREST 0.00000000 sec
MCWRK 0.01500000 sec
======== CHANNEL f1 ========
NUC1 13C
P1 8.70 usec
PL1 -5.00 dB
SFO1 100.6278209 MHz
======== CHANNEL f2 ========
CPDPRG2 waltz16
NUC2 1H
PCPD2 80.00 usec
PL2 -4.00 dB
PL12 11.78 dB
SFO2 400.1320007 MHz
F2 - Processing parameters
SI 32768
SF 100.6127849 MHz
WDW EM
SSB 0
LB 1.00 Hz
GB 0
PC 1.40
1D NMR plot parameters
CX 25.00 cm
CY 11.61 cm
F1P 150.000 ppm
F1 15091.92 Hz
F2P -10.000 ppm
F2 -1006.13 Hz
PPMCM 6.40000 ppm/cm
HZCM 643.92181 Hz/cm
81.09
75.02
71.68
65.49
49.64
49.42
49.21
49.00
48.79
48.57
48.36
24.99
OH
HO
OH
120 115 110 105 100 95 90 85 80 75 70 65 60 55 50 45 40 35 30 25 20 15 10 5

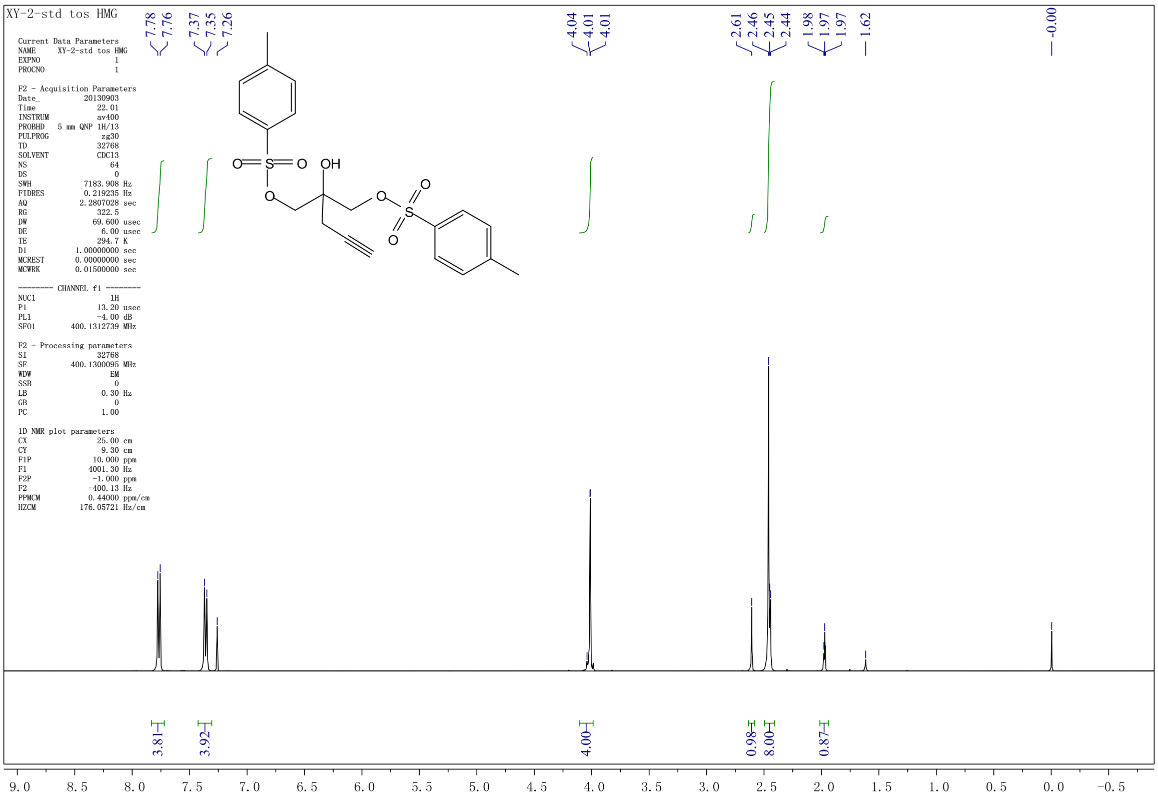
XY-2-std tos HMG
Current Data Parameters
NAME XY-2-std tos HMG
EXPNO 1
PROCNO 1
F2 - Acquisition Parameters
Date_ 20130903
Time 22.01
INSTRUM av400
PROBHD 5 mm QNP 1H/13
PULPROG zg30
TD 32768
SOLVENT CDCl3
NS 64
DS 0
SWH 7183.908 Hz
FIDRES 0.219235 Hz
AQ 2.2807028 sec
RG 322.5
DW 69.600 usec
DE 6.00 usec
TE 294.7 K
D1 1.00000000 sec
MCREST 0.00000000 sec
MCWRK 0.01500000 sec
======== CHANNEL f1 ========
NUC1 1H
P1 13.20 usec
PL1 -4.00 dB
SFO1 400.1312739 MHz
F2 - Processing parameters
SI 32768
SF 400.1300095 MHz
WDW EM
SSB 0
LB 0.30 Hz
GB 0
PC 1.00
1D NMR plot parameters
CX 25.00 cm
CY 9.30 cm
F1P 10.000 ppm
F1 4001.30 Hz
F2P -1.000 ppm
F2 -400.13 Hz
PPMCM 0.44000 ppm/cm
HZCM 176.05721 Hz/cm
7.78 7.76 7.37 7.35 7.26 4.04 4.01 4.01 2.61 2.46 2.45 2.44 1.98 1.97 1.97 1.62 -0.00
O S O OH O O S O
3.81 3.92 4.00 0.98 8.00 0.87
9.0 8.5 8.0 7.5 7.0 6.5 6.0 5.5 5.0 4.5 4.0 3.5 3.0 2.5 2.0 1.5 1.0 0.5 0.0 -0.5

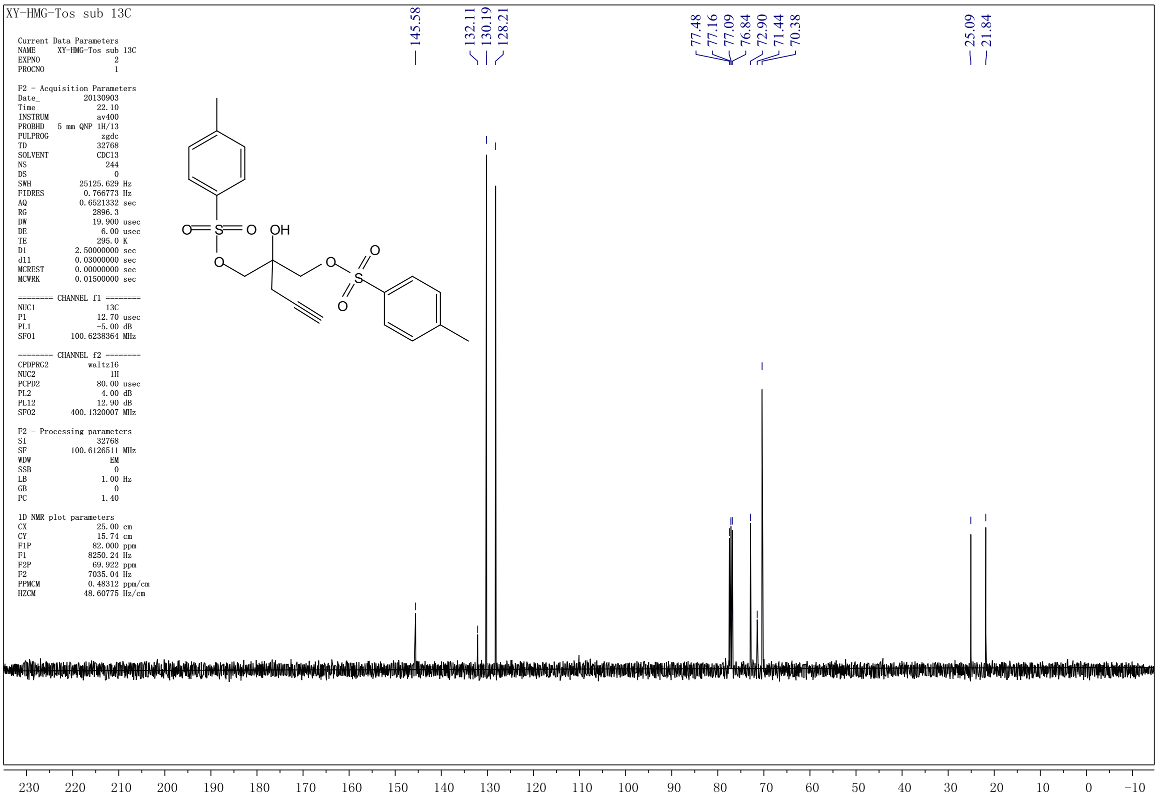
XY-HMG-Tos sub 13C
Current Data Parameters
NAME XY-HMG-Tos sub 13C
EXPNO 2
PROCNO 1
F2 - Acquisition Parameters
Date_ 20130903
Time 22.10
INSTRUM av400
PROBHD 5 mm QNP 1H/13
PULPROG zgdc
TD 32768
SOLVENT CDCl3
NS 244
DS 0
SWH 25125.629 Hz
FIDRES 0.766773 Hz
AQ 0.6521332 sec
RG 2896.3
DW 19.900 usec
DE 6.00 usec
TE 295.0 K
D1 2.50000000 sec
d11 0.03000000 sec
MCREST 0.00000000 sec
MCWRK 0.01500000 sec
======== CHANNEL f1 ========
NUC1 13C
P1 12.70 usec
PL1 -5.00 dB
SFO1 100.6238364 MHz
======== CHANNEL f2 ========
CPDPRG2 waltz16
NUC2 1H
PCPD2 80.00 usec
PL2 -4.00 dB
PL12 12.90 dB
SFO2 400.1320007 MHz
F2 - Processing parameters
SI 32768
SF 100.6126511 MHz
WDW EM
SSB 0
LB 1.00 Hz
GB 0
PC 1.40
1D NMR plot parameters
CX 25.00 cm
CY 15.74 cm
F1P 82.000 ppm
F1 8250.24 Hz
F2P 69.922 ppm
F2 7035.04 Hz
PPMCM 0.48312 ppm/cm
HZCM 48.60775 Hz/cm
145.58 132.11 130.19 128.21 77.48 77.16 77.09 76.84 72.90 71.44 70.38 25.09 21.84
O S O OH O O S O
230 220 210 200 190 180 170 160 150 140 130 120 110 100 90 80 70 60 50 40 30 20 10 0 -10

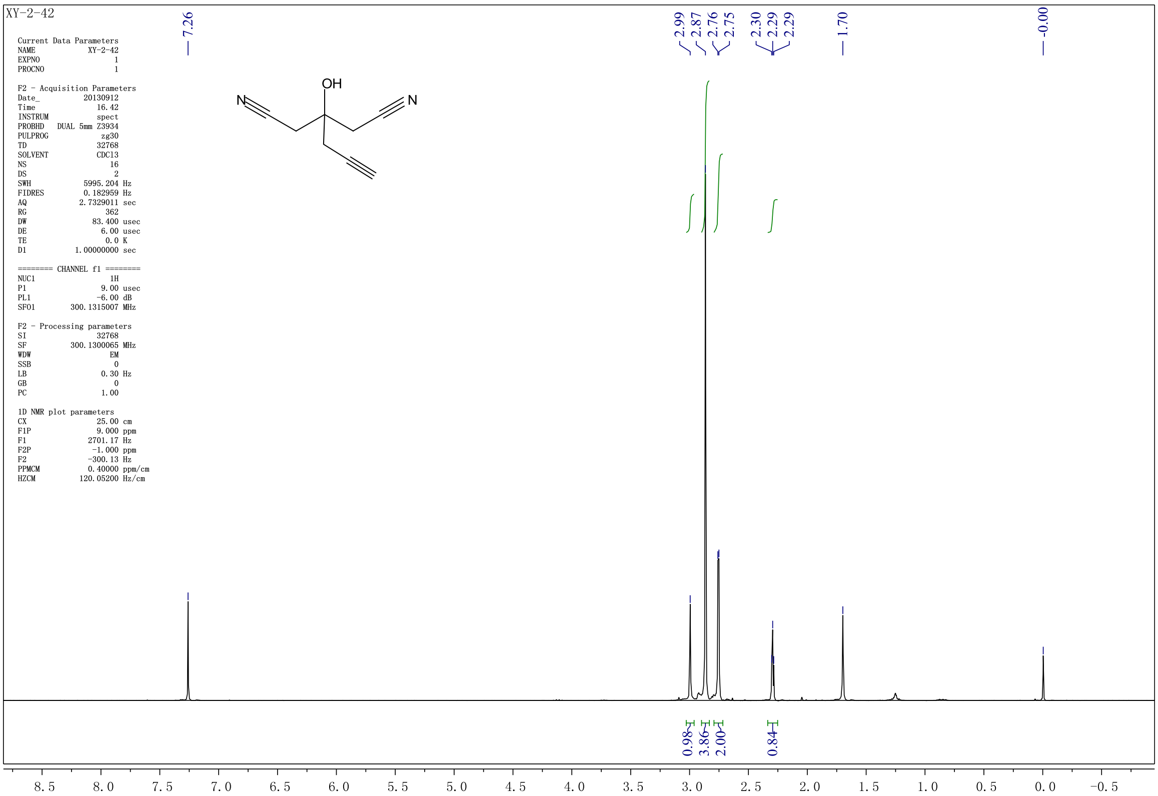
XY-2-42
Current Data Parameters
NAME XY-2-42
EXPNO 1
PROCNO 1
F2 - Acquisition Parameters
Date_ 20130912
Time 16.42
INSTRUM spect
PROBHD DUAL 5mm Z3934
PULPROG zg30
TD 32768
SOLVENT CDCl3
NS 16
DS 2
SWH 5995.204 Hz
FIDRES 0.182959 Hz
AQ 2.7329011 sec
RG 362
DW 83.400 usec
DE 6.00 usec
TE 0.0 K
D1 1.00000000 sec
======== CHANNEL f1 ========
NUC1 1H
P1 9.00 usec
PL1 -6.00 dB
SFO1 300.1315007 MHz
F2 - Processing parameters
SI 32768
SF 300.1300065 MHz
WDW EM
SSB 0
LB 0.30 Hz
GB 0
PC 1.00
1D NMR plot parameters
CX 25.00 cm
F1P 9.000 ppm
F1 2701.17 Hz
F2P -1.000 ppm
F2 -300.13 Hz
PPMCM 0.40000 ppm/cm
HZCM 120.05200 Hz/cm
OH
N
N
7.26
2.99
2.87
2.76
2.75
2.30
2.29
2.29
1.70
-0.00
0.98
3.86
2.00
0.84
8.5 8.0 7.5 7.0 6.5 6.0 5.5 5.0 4.5 4.0 3.5 3.0 2.5 2.0 1.5 1.0 0.5 0.0 -0.5

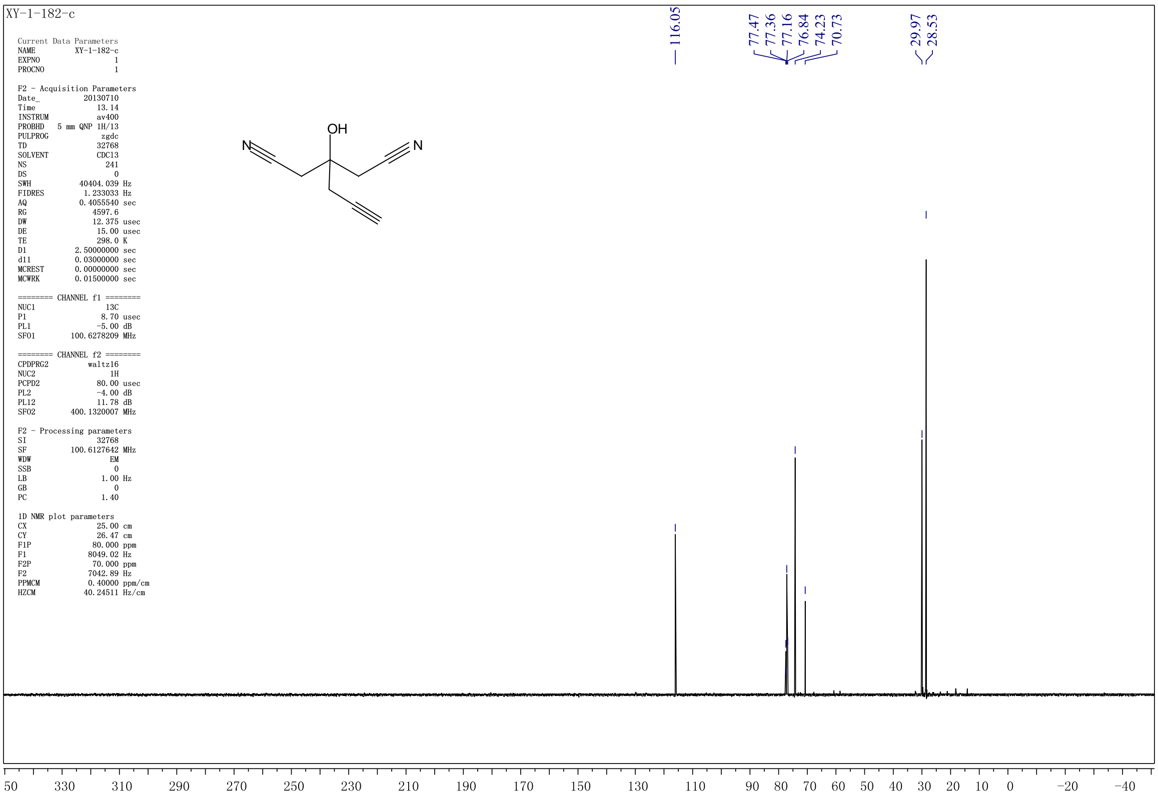
XY-1-182-c
Current Data Parameters
NAME XY-1-182-c
EXPNO 1
PROCNO 1
F2 - Acquisition Parameters
Date_ 20130710
Time 13.14
INSTRUM av400
PROBHD 5 mm QNP 1H/13
PULPROG zgdc
TD 32768
SOLVENT CDCl3
NS 241
DS 0
SWH 40404.039 Hz
FIDRES 1.233033 Hz
AQ 0.4055540 sec
RG 4597.6
DW 12.375 usec
DE 15.00 usec
TE 298.0 K
D1 2.50000000 sec
d11 0.03000000 sec
MCREST 0.00000000 sec
MCWRK 0.01500000 sec
======== CHANNEL f1 ========
NUC1 13C
P1 8.70 usec
PL1 -5.00 dB
SFO1 100.6278209 MHz
======== CHANNEL f2 ========
CPDPRG2 waltz16
NUC2 1H
PCPD2 80.00 usec
PL2 -4.00 dB
PL12 11.78 dB
SFO2 400.1320007 MHz
F2 - Processing parameters
SI 32768
SF 100.6127642 MHz
WDW EM
SSB 0
LB 1.00 Hz
GB 0
PC 1.40
1D NMR plot parameters
CX 25.00 cm
CY 26.47 cm
F1P 80.000 ppm
F1 8049.02 Hz
F2P 70.000 ppm
F2 7042.89 Hz
PPMCM 0.40000 ppm/cm
HZCM 40.24511 Hz/cm
OH
N
N
116.05
77.47
77.36
77.16
76.84
74.23
70.73
29.97
28.53
50 330 310 290 270 250 230 210 190 170 150 130 110 90 80 70 60 50 40 30 20 10 0 -20 -40

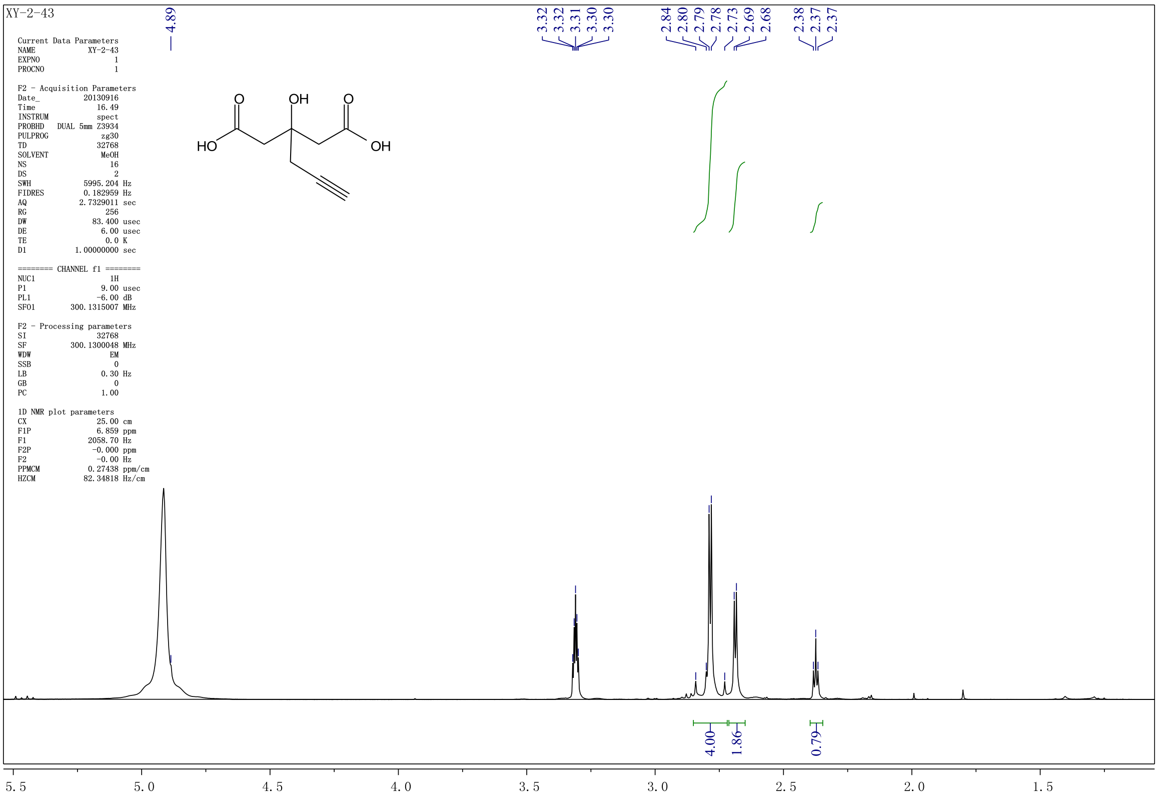
XY-2-43
Current Data Parameters
NAME XY-2-43
EXPNO 1
PROCNO 1
F2 - Acquisition Parameters
Date_ 20130916
Time 16.49
INSTRUM spect
PROBHD DUAL 5mm Z3934
PULPROG zg30
TD 32768
SOLVENT MeOH
NS 16
DS 2
SWH 5995.204 Hz
FIDRES 0.182959 Hz
AQ 2.7329011 sec
RG 256
DW 83.400 usec
DE 6.00 usec
TE 0.0 K
D1 1.00000000 sec
======= CHANNEL f1 =======
NUC1 1H
P1 9.00 usec
PL1 -6.00 dB
SFO1 300.1315007 MHz
F2 - Processing parameters
SI 32768
SF 300.1300048 MHz
WDW EM
SSB 0
LB 0.30 Hz
GB 0
PC 1.00
1D NMR plot parameters
CX 25.00 cm
F1P 6.859 ppm
F1 2058.70 Hz
F2P -0.000 ppm
F2 -0.00 Hz
PPMCM 0.27438 ppm/cm
HZCM 82.34818 Hz/cm
4.89
3.32 3.32 3.31 3.30 3.30
2.84 2.80 2.79 2.78 2.73 2.69 2.68
2.38 2.37 2.37
O OH O
HO OH
4.00 1.86 0.79
5.5 5.0 4.5 4.0 3.5 3.0 2.5 2.0 1.5

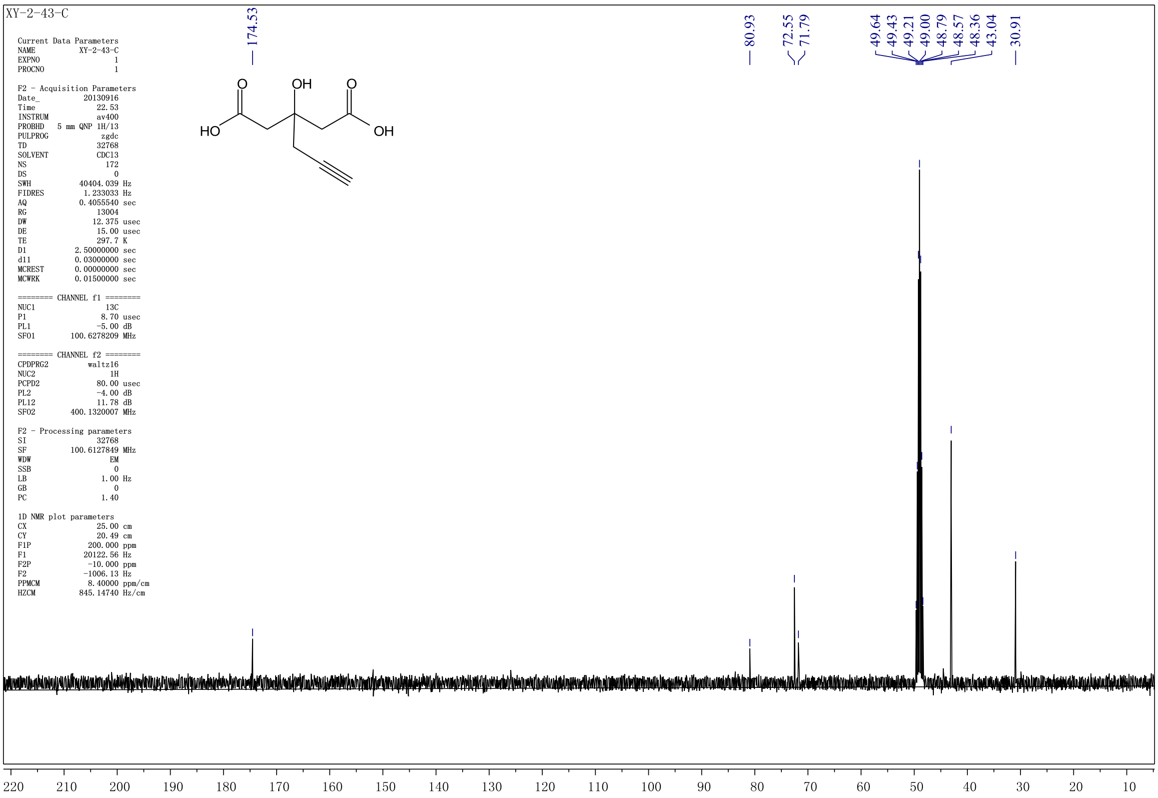
XY-2-43-C
Current Data Parameters
NAME XY-2-43-C
EXPNO 1
PROCNO 1
F2 - Acquisition Parameters
Date_ 20130916
Time 22.53
INSTRUM av400
PROBHD 5 mm QNP 1H/13
PULPROG zgdc
TD 32768
SOLVENT CDCl3
NS 172
DS 0
SWH 40404.039 Hz
FIDRES 1.233033 Hz
AQ 0.4055540 sec
RG 13004
DW 12.375 usec
DE 15.00 usec
TE 297.7 K
D1 2.50000000 sec
d11 0.03000000 sec
MCREST 0.00000000 sec
MCWRK 0.01500000 sec
======= CHANNEL f1 =======
NUC1 13C
P1 8.70 usec
PL1 -5.00 dB
SFO1 100.6278209 MHz
======= CHANNEL f2 =======
CPDPRG2 waltz16
NUC2 1H
PCPD2 80.00 usec
PL2 -4.00 dB
PL12 11.78 dB
SFO2 400.1320007 MHz
F2 - Processing parameters
SI 32768
SF 100.6127849 MHz
WDW EM
SSB 0
LB 1.00 Hz
GB 0
PC 1.40
1D NMR plot parameters
CX 25.00 cm
CY 20.49 cm
F1P 200.000 ppm
F1 20122.56 Hz
F2P -10.000 ppm
F2 -1006.13 Hz
PPMCM 8.40000 ppm/cm
HZCM 845.14740 Hz/cm
174.53
80.93
72.55 71.79
49.64 49.43 49.21 49.00 48.79 48.57 48.36 43.04
30.91
O OH O
HO OH
220 210 200 190 180 170 160 150 140 130 120 110 100 90 80 70 60 50 40 30 20 10

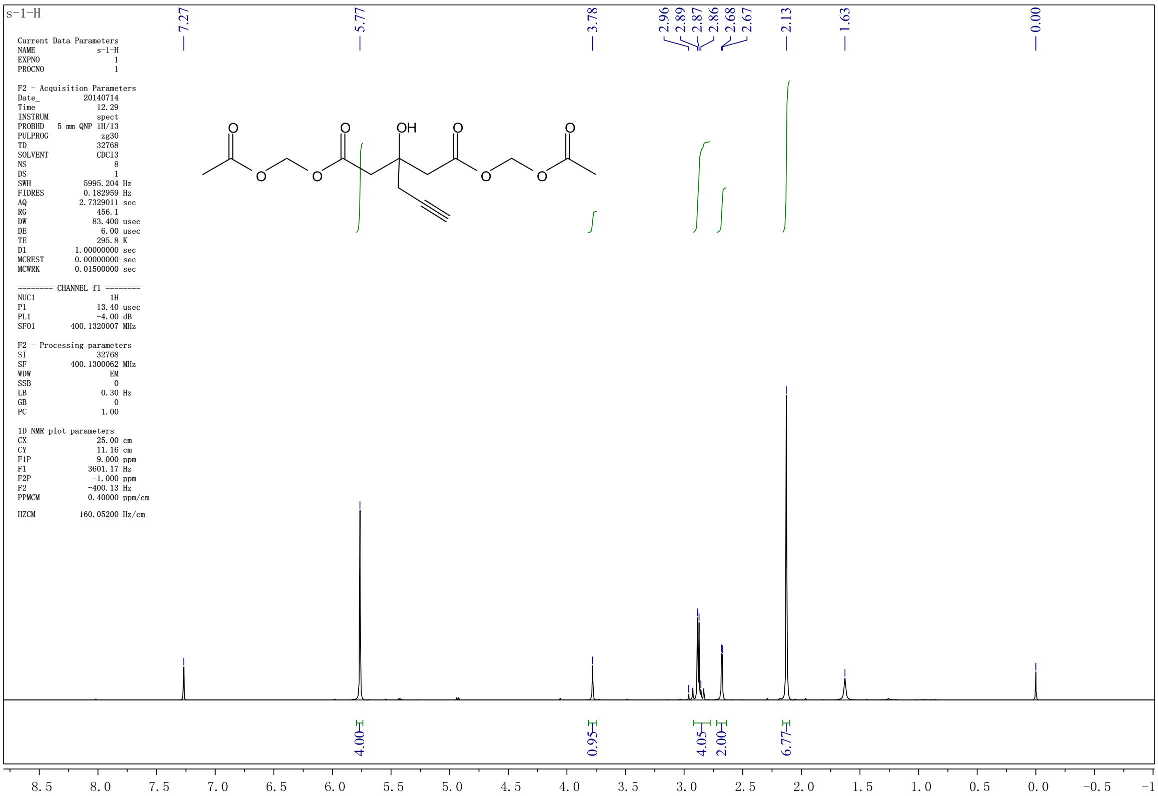

s-1-H
7.27
5.77
3.78
2.96
2.89
2.87
2.86
2.68
2.67
2.13
1.63
0.00
Current Data Parameters
NAME s-1-H
EXPNO 1
PROCNO 1
F2 - Acquisition Parameters
Date_ 20140714
Time 12.29
INSTRUM spect
PROBHD 5 mm QNP 1H/13
PULPROG zg30
TD 32768
SOLVENT CDCl3
NS 8
DS 1
SWH 5995.204 Hz
FIDRES 0.182959 Hz
AQ 2.7329011 sec
RG 456.1
DW 83.400 usec
DE 6.00 usec
TE 295.8 K
D1 1.00000000 sec
MCREST 0.00000000 sec
MCWRK 0.01500000 sec
======== CHANNEL f1 ========
NUC1 1H
P1 13.40 usec
PL1 -4.00 dB
SFO1 400.1320007 MHz
F2 - Processing parameters
SI 32768
SF 400.1300062 MHz
WDW EM
SSB 0
LB 0.30 Hz
GB 0
PC 1.00
1D NMR plot parameters
CX 25.00 cm
CY 11.16 cm
F1P 9.000 ppm
F1 3601.17 Hz
F2P -1.000 ppm
F2 -400.13 Hz
PPMCM 0.40000 ppm/cm
HZCM 160.05200 Hz/cm
O
O
OH
O
O
O
O
O
O
4.00
0.95
4.05
2.00
6.77
8.5
8.0
7.5
7.0
6.5
6.0
5.5
5.0
4.5
4.0
3.5
3.0
2.5
2.0
1.5
1.0
0.5
0.0
-0.5
-1


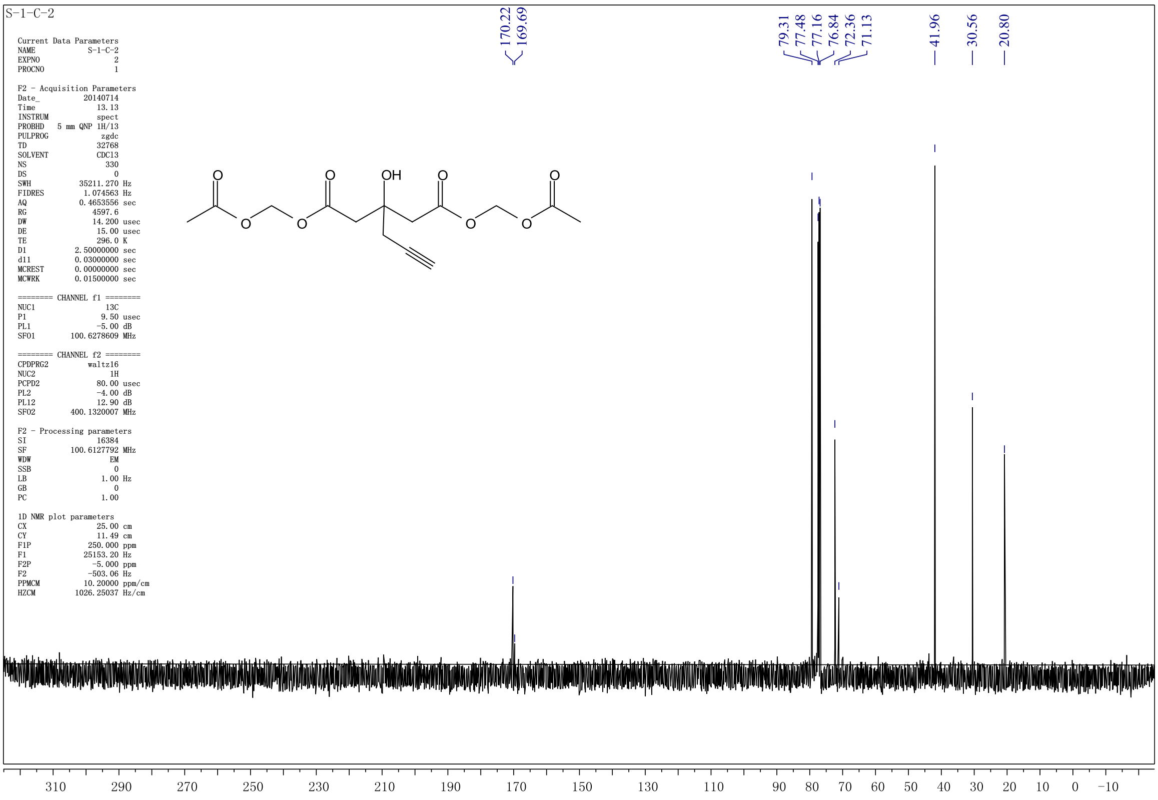

S-1-C-2
170.22
169.69
79.31
77.48
77.16
76.84
72.36
71.13
41.96
30.56
20.80
Current Data Parameters
NAME S-1-C-2
EXPNO 2
PROCNO 1
F2 - Acquisition Parameters
Date_ 20140714
Time 13.13
INSTRUM spect
PROBHD 5 mm QNP 1H/13
PULPROG zgdc
TD 32768
SOLVENT CDCl3
NS 330
DS 0
SWH 35211.270 Hz
FIDRES 1.074563 Hz
AQ 0.4653556 sec
RG 4597.6
DW 14.200 usec
DE 15.00 usec
TE 296.0 K
D1 2.50000000 sec
d11 0.03000000 sec
MCREST 0.00000000 sec
MCWRK 0.01500000 sec
======== CHANNEL f1 ========
NUC1 13C
P1 9.50 usec
PL1 -5.00 dB
SFO1 100.6278609 MHz
======== CHANNEL f2 ========
CPDPRG2 waltz16
NUC2 1H
PCPD2 80.00 usec
PL2 -4.00 dB
PL12 12.90 dB
SFO2 400.1320007 MHz
F2 - Processing parameters
SI 16384
SF 100.6127792 MHz
WDW EM
SSB 0
LB 1.00 Hz
GB 0
PC 1.00
1D NMR plot parameters
CX 25.00 cm
CY 11.49 cm
F1P 250.000 ppm
F1 25153.20 Hz
F2P -5.000 ppm
F2 -503.06 Hz
PPMCM 10.20000 ppm/cm
HZCM 1026.25037 Hz/cm
O
O
OH
O
O
O
O
O
O
310
290
270
250
230
210
190
170
150
130
110
90
80
70
60
50
40
30
20
10
0
-10

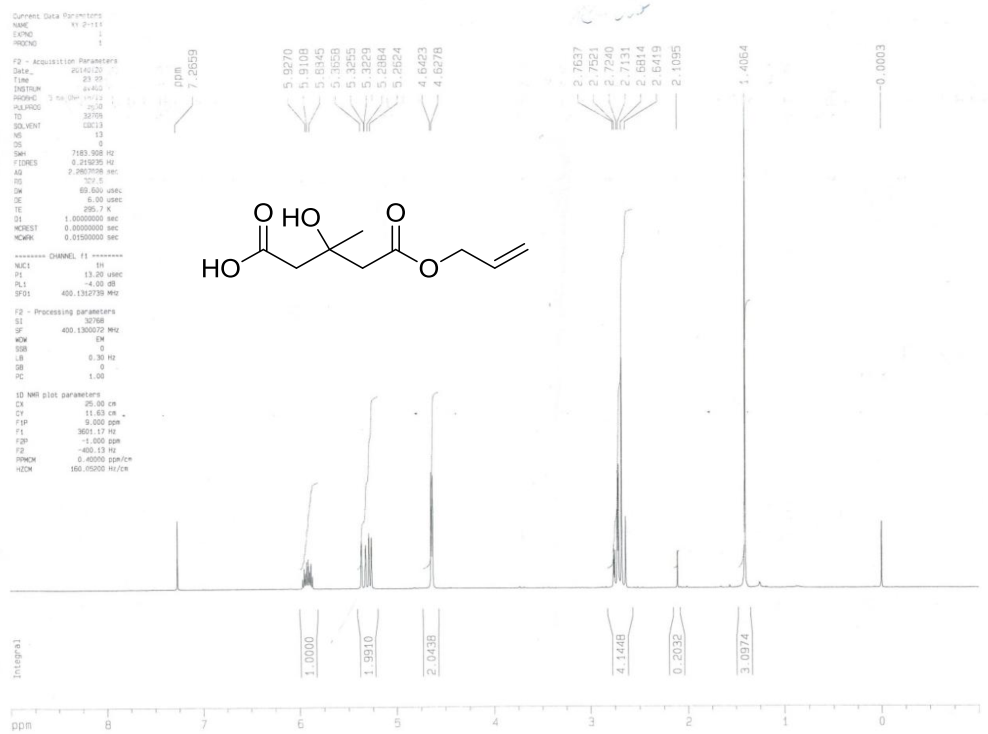

7.2659
5.9270
5.9108
5.8345
5.3658
5.3255
5.3229
5.2884
5.2624
4.6423
4.6278
2.7637
2.7521
2.7240
2.7131
2.6814
2.6419
2.1095
1.4064
0.0003
HO
O
O
HO
O
1.0000
1.9910
2.0438
4.1448
0.2032
3.0974
Integral
ppm


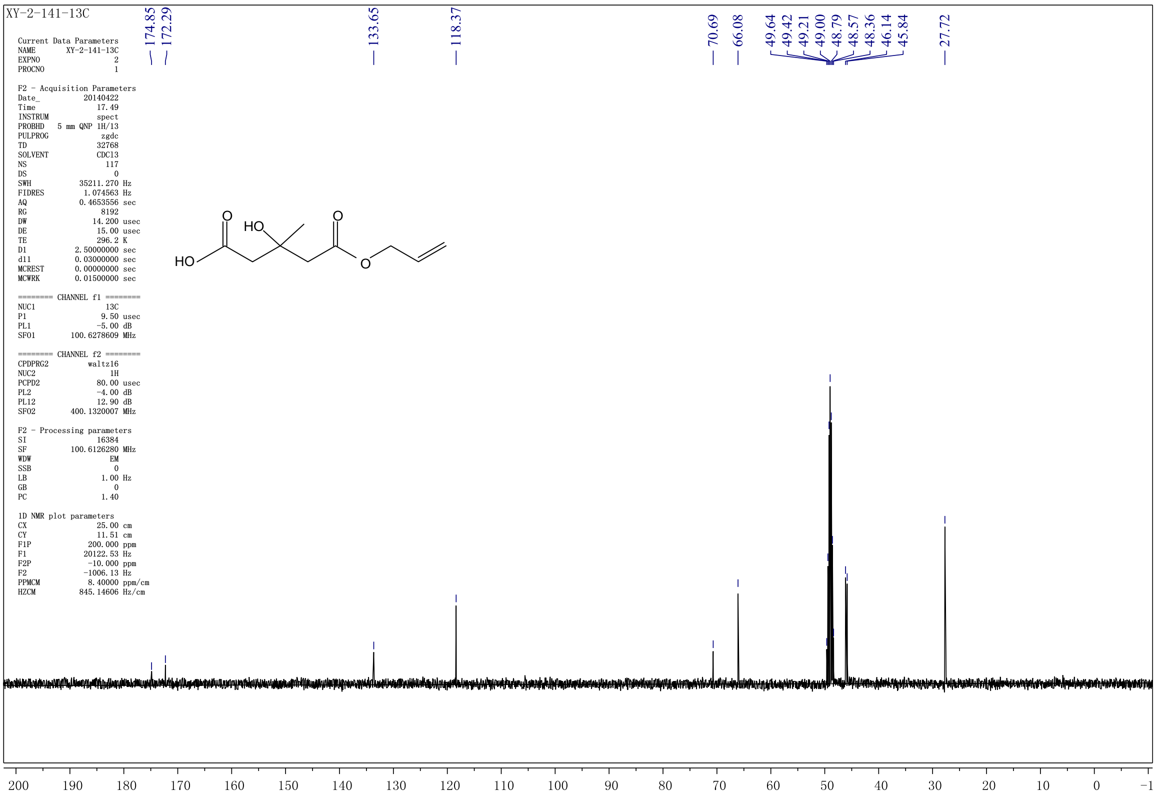

XY-2-141-13C
174.85
172.29
133.65
118.37
70.69
66.08
49.64
49.42
49.21
49.00
48.79
48.57
48.36
46.14
45.84
27.72
Current Data Parameters
NAME XY-2-141-13C
EXPNO 2
PROCNO 1
F2 - Acquisition Parameters
Date_ 20140422
Time 17.49
INSTRUM spect
PROBHD 5 mm QNP 1H/13
PULPROG zgdc
TD 32768
SOLVENT CDCl3
NS 117
DS 0
SWH 35211.270 Hz
FIDRES 1.074563 Hz
AQ 0.4653556 sec
RG 8192
DW 14.200 usec
DE 15.00 usec
TE 296.2 K
D1 2.50000000 sec
d11 0.03000000 sec
MCREST 0.00000000 sec
MCWRK 0.01500000 sec
======== CHANNEL f1 ========
NUC1 13C
P1 9.50 usec
PL1 -5.00 dB
SFO1 100.6278609 MHz
======== CHANNEL f2 ========
CPDPRG2 waltz16
NUC2 1H
PCPD2 80.00 usec
PL2 -4.00 dB
PL12 12.90 dB
SFO2 400.1320007 MHz
F2 - Processing parameters
SI 16384
SF 100.6126280 MHz
WDW EM
SSB 0
LB 1.00 Hz
GB 0
PC 1.40
1D NMR plot parameters
CX 25.00 cm
CY 11.51 cm
F1P 200.000 ppm
F1 20122.53 Hz
F2P -10.000 ppm
F2 -1006.13 Hz
PPMCM 8.40000 ppm/cm
HZCM 845.14606 Hz/cm
HO
O
O
HO
O

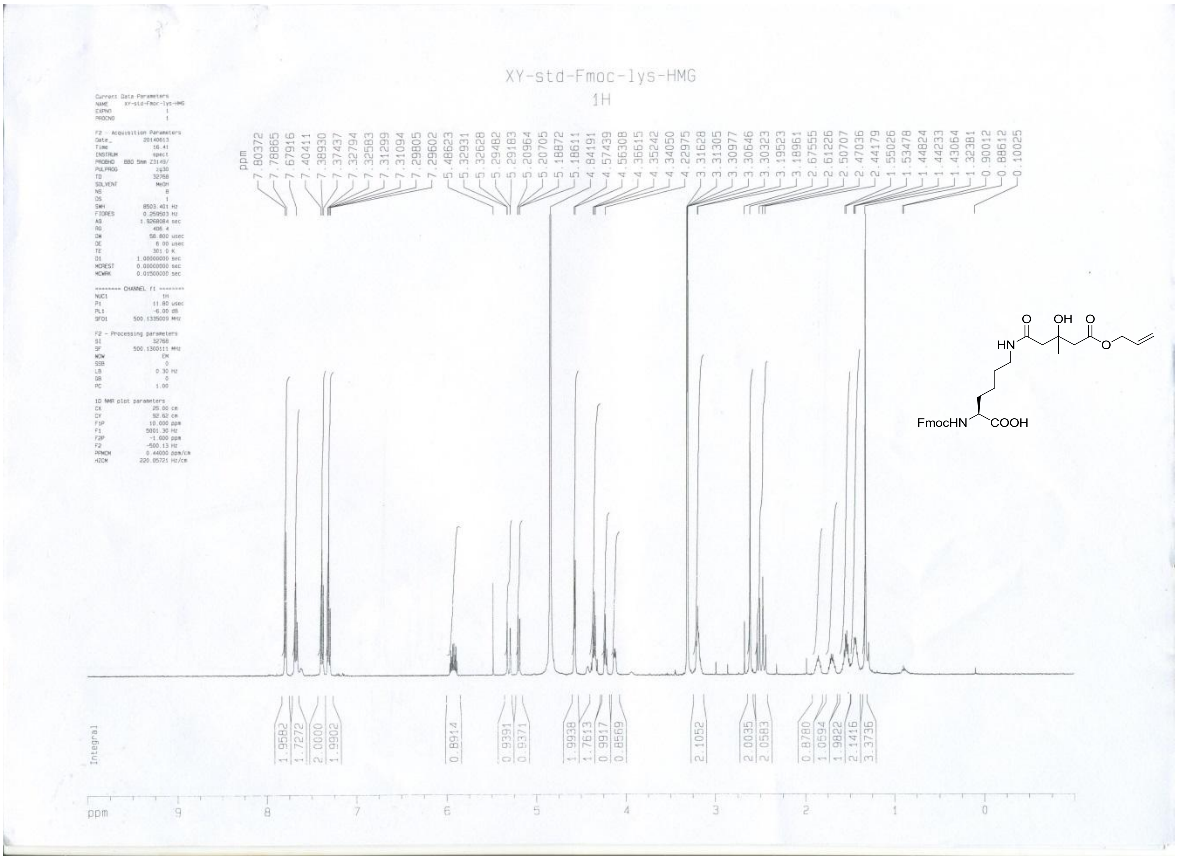
XY-std-Fmoc-lys-HMG
1H
ppm
7.80372
7.78865
7.67916
7.40411
7.38930
7.37437
7.32794
7.32583
7.31299
7.31094
7.29805
7.29602
5.48623
5.32931
5.32628
5.29482
5.29183
5.20964
5.20705
5.18872
5.18611
4.84191
4.57439
4.56308
4.36615
4.35242
4.34050
4.22975
3.31628
3.31305
3.30977
3.30646
3.30323
3.19523
3.18961
2.67555
2.61226
2.50707
2.47036
2.44179
1.55026
1.53478
1.44824
1.44233
1.43064
1.32381
0.90012
0.88612
0.10025
HN
O
OH
O
O
FmocHN
COOH
Integral
1.9582
1.7272
2.0000
1.9902
0.8914
0.9391
0.9371
1.9938
1.7613
0.9917
0.8569
2.1052
2.0035
2.0583
0.8780
1.0594
1.9822
2.1416
3.3736
ppm
9
8
7
6
5
4
3
2
1
0

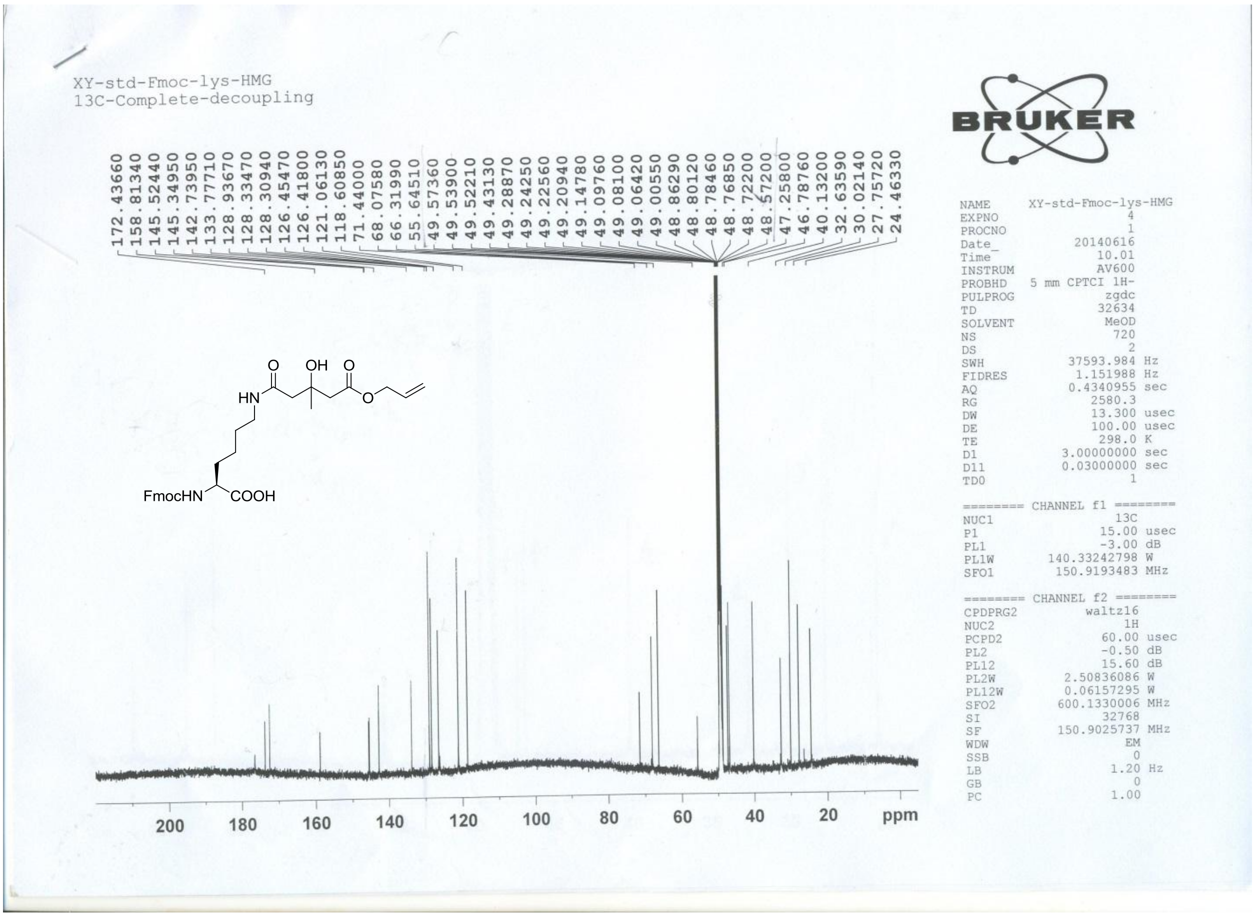
XY-std-Fmoc-lys-HMG
13C-Complete-decoupling
BRUKER
172.43660
158.81340
145.52440
145.34950
142.73950
133.77710
128.93670
128.33470
128.30940
126.45470
126.41800
121.06130
118.60850
71.44000
68.07580
66.31990
55.64510
49.57360
49.53900
49.52210
49.43130
49.28870
49.22560
49.20940
49.14780
49.09760
49.08100
49.06420
49.00550
48.86290
48.80120
48.78460
48.76850
48.72200
48.57200
47.25800
46.78760
40.13200
32.63590
30.02140
27.75720
24.46330
HN
O
OH
O
O
FmocHN
COOH
200
180
160
140
120
100
80
60
40
20
ppm
NAME XY-std-Fmoc-lys-HMG
EXPNO 4
PROCNO 1
Date_ 20140616
Time 10.01
INSTRUM AV600
PROBHD 5 mm CPTCI 1H-
PULPROG zgdc
TD 32634
SOLVENT MeOD
NS 720
DS 2
SWH 37593.984 Hz
FIDRES 1.151988 Hz
AQ 0.4340955 sec
RG 2580.3
DW 13.300 usec
DE 100.00 usec
TE 298.0 K
D1 3.00000000 sec
D11 0.03000000 sec
TD0 1
======== CHANNEL f1 ========
NUC1 13C
P1 15.00 usec
PL1 -3.00 dB
PL1W 140.33242798 W
SFO1 150.9193483 MHz
======== CHANNEL f2 ========
CPDPRG2 waltz16
NUC2 1H
PCPD2 60.00 usec
PL2 -0.50 dB
PL12 15.60 dB
PL2W 2.50836086 W
PL12W 0.06157295 W
SFO2 600.1330006 MHz
SI 32768
SF 150.9025737 MHz
WDW EM
SSB 0
LB 1.20 Hz
GB 0
PC 1.00

## Supplementary References


[1] M. R. Wieckowski, C. Giorgi, M. Lebiedzinska, J. Duszynski, P. Pinton, *Nature protocols* **2009**, *4*, 1582-1590.

[2] D. Shechter, H. L. Dormann, C. D. Allis, S. B. Hake, *Nature protocols* **2007**, *2*, 1445-1457.

[3] M. Tan, H. Luo, S. Lee, F. Jin, J. S. Yang, E. Montellier, T. Buchou, Z. Cheng, S. Rousseaux, N. Rajagopal, Z. Lu, Z. Ye, Q. Zhu, J. Wysocka, Y. Ye, S. Khochbin, B. Ren, Y. Zhao, *Cell* **2011**, *146*, 1016-1028.

[4] K. V. Baratha, A. Nourry, J. F. Pilard, *Eur Polym J* **2015**, *70*, 317-330.

[5] J. Zhou, Y. Y. Yeung, *J Org Chem* **2014**, *79*, 4644-4649.
